# Determining Sequence-Dependent DNA Oligonucleotide Hybridization and Dehybridization Mechanisms Using Coarse-Grained Molecular Simulation, Markov State Models, and Infrared Spectroscopy

**DOI:** 10.1021/jacs.1c05219

**Published:** 2021-10-13

**Authors:** Michael S. Jones, Brennan Ashwood, Andrei Tokmakoff, Andrew L. Ferguson

**Affiliations:** † Pritzker School of Molecular Engineering, The University of Chicago, 5640 South Ellis Avenue, Chicago, Illinois 60637, United States; ‡ Department of Chemistry, Institute for Biophysical Dynamics, and James Franck Institute, The University of Chicago, 929 East 57th Street, Chicago, Illinois 60637, United States

## Abstract

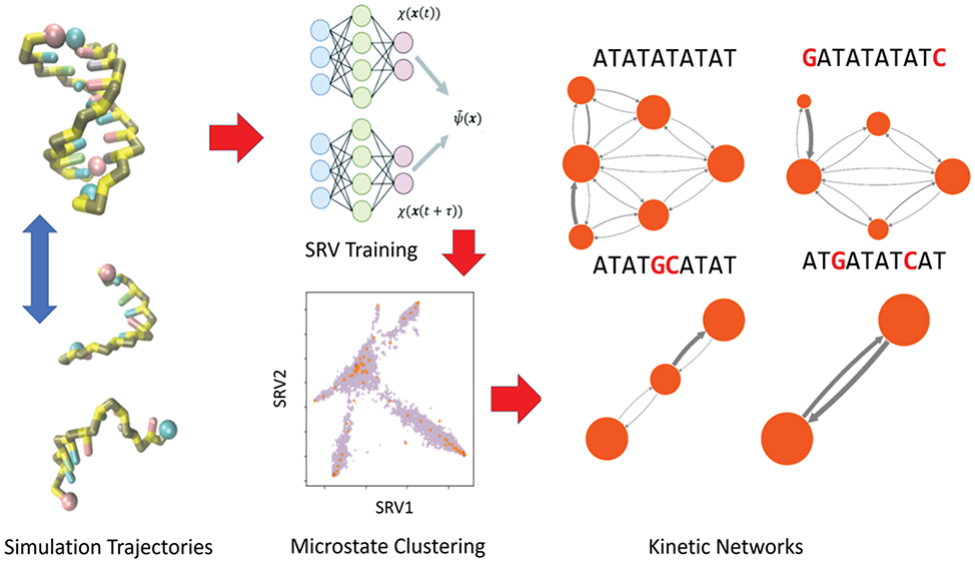

A robust understanding of the sequence-dependent thermodynamics of DNA hybridization has enabled rapid advances in DNA nanotechnology. A fundamental understanding of the sequence-dependent kinetics and mechanisms of hybridization and dehybridization remains comparatively underdeveloped. In this work, we establish new understanding of the sequence-dependent hybridization/dehybridization kinetics and mechanism within a family of self-complementary pairs of 10-mer DNA oligomers by integrating coarse-grained molecular simulation, machine learning of the slow dynamical modes, data-driven inference of long-time kinetic models, and experimental temperature-jump infrared spectroscopy. For a repetitive ATATATATAT sequence, we resolve a rugged dynamical landscape comprising multiple metastable states, numerous competing hybridization/dehybridization pathways, and a spectrum of dynamical relaxations. Introduction of a G:C pair at the terminus (GATATATATC) or center (ATATGCATAT) of the sequence reduces the ruggedness of the dynamics landscape by eliminating a number of metastable states and reducing the number of competing dynamical pathways. Only by introducing a G:C pair midway between the terminus and the center to maximally disrupt the repetitive nature of the sequence (ATGATATCAT) do we recover a canonical “all-or-nothing” two-state model of hybridization/dehybridization with no intermediate metastable states. Our results establish new understanding of the dynamical richness of sequence-dependent kinetics and mechanisms of DNA hybridization/dehybridization by furnishing quantitative and predictive kinetic models of the dynamical transition network between metastable states, present a molecular basis with which to understand experimental temperature jump data, and furnish foundational design rules by which to rationally engineer the kinetics and pathways of DNA association and dissociation for DNA nanotechnology applications.

## 1 Introduction

Over the last couple of decades, DNA has proven to be much more than a vessel for genetic information. From sensing to computing to directed self-assembly, the programmable and predictable nature of DNA has unlocked numerous unforeseen nanotechnology applications.^1−4^ Recently, single molecule localization techniques have exploited the rapid and transient binding of short DNA oligomers in order to achieve super-resolution microscopy and optical multiplexing.^5−7^ Predictive understanding of the sequence-dependent thermodynamics of DNA hybridization/dehybridization—the assembly/disassembly of a DNA duplex from two single strands—has underpinned the rational design of DNA oligomer sequences for nanotechnology applications, where sequence-dependent nearest-neighbor models can accurately account for mismatched pairs, dangling ends, and other non-native bonding effects.^8,9^ Secondary DNA structures such as hairpins and G-quadruplexes have also been studied in depth and leveraged for nanotechnology applications.^10−12^ Predictive models of the dynamical, as opposed to purely thermodynamical, behaviors of DNA have become increasingly important in developing technologies such as DNA-PAINT (DNA Points Accumulation for Imaging in Nanoscale Topography), but these technologies have outpaced our fundamental understanding of the dynamics themselves.^13−15^ Many experimental and computational studies have investigated DNA dynamical phenomena over picosecond to millisecond time scales.^16−20^ Kinetic models have been developed for particular DNA processes such as toehold exchanges and optical barcoding^21,22^ and supervised machine learning techniques have been combined with experimental measurements to predict the on/off rates as a function of sequence.^6,23,24^ A comprehensive understanding of the full dynamical landscape of hybridization/dehybridization accounting for the sequence-dependent metastable states and association/dissociation pathways remains lacking and fundamental questions remain unresolved. For example, it remains unclear the extent to which hybridization of short DNA oligomers largely proceeds in a conventionally assumed “all-or-nothing” fashion or if long-lived metastable states facilitate the transition.^17,19,25−28^ Out-of-register “shifted” base-paired structures^17,18,25,29−31^ and frayed structures^32−35^ stand as candidates for metastable states with the potential to mediate substantial deviations from all-or-nothing behavior, but the degree to which these states are kinetically relevant is difficult to determine experimentally and is likely to be highly sequence-dependent. The development of predictive models and design rules with which to engineer DNA strands with tailored hybridization/dehybridization kinetics and pathways is vital to advancing rational design of DNA strands for nanotechnology applications and is also of importance in understanding fundamental biological processes such as transcription and gene regulation.

Our understanding of hybridization dynamics is built upon decades of experiments—such as temperature-jump, salt-jump, pH-jump, and other perturbative methods—that rapidly stimulate DNA and monitor relaxation to a new equilibrium.^28,36−42^ More recently, single-molecule diffusion and tethered multi-fluorophore assays have facilitated analyses under equilibrium conditions, but these results can be hampered by slow data collection rates and fluorescent tags effects on strand dynamics, particularly for shorter oligomers.^23,43−45^ A number of computational modeling approaches have also been employed to provide molecular-level resolution of hybridization. Simplified lattice models can recapitulate the essential aspects of the hybridization pathways but lack the realism of continuous space representations.^25,46^ The long time scales associated with hybridization/dehybridization events place them outside the reach of unbiased all-atom molecular dynamics simulations,^18^ but they can be observed by employing enhanced sampling techniques^18,30,47−53^ or by using elevated temperature or denaturing solvent concentrations to induce one-way dissociation events.^54,55^ The effect of the applied bias upon the thermodynamics can be rigorously corrected for using standard reweighting techniques.^56−59^ Rigorous elimination of the bias in the kinetics is critical for the construction of robust and reliable kinetic models that reflect the true system dynamics and are uncontaminated by any residual effects of the biasing potentials used to induce good sampling. A number of approaches to correct the kinetics are also available, including Girsanov reweighting, transition-based reweighting analysis (TRAM), dynamic histogram analysis method (DHAM), and their derivatives.^60−66^ The application of these methods under the conditions of high bias necessary for good sampling can, however, present challenges for numerical convergence. A number of coarse-grained DNA force fields have been developed that enable direct observation of these events over microsecond time scales via unbiased coarse-grained molecular dynamics simulations,^29,30,49,67,68^ which, up to an acceleration factor associated with the smoothing of the underlying free energy landscape inherent to the coarse-graining procedure, can preserve a faithful model of the unbiased dynamics and associated pathways. These models have previously been used to study biological phenomena such as nucleosome dynamics^69,70^ and transcription factor binding^71,72^ as well as nanotechnology applications such as strand displacement^73,74^ and DNA origami.^75,76^ In this study, we choose to employ a coarse-grained model for DNA^49^ that is sufficiently inexpensive to enable the collection of sufficient volumes of unbiased simulation trajectories and adequately sample configurational space that we do not need to appeal to biasing strategies to enhance convergence nor apply any *post hoc* corrections to the thermodynamics or kinetics.

In this work, we study a family of self-complementary pairs of 10-mer DNA oligomers using coarse-grained molecular simulation, machine learning of the slow dynamical modes, and data-driven inference of long-time kinetic models to establish new understanding of the influence of sequence upon hybridization/dehybridization kinetics and mechanisms. This family—5′-ATATATATAT-3′ (AT-all), 5′-GATATATATC-3′ (GC-end), 5′-ATATGCATAT-3′ (GC-core), and 5′-ATGATATCAT-3′ (GC-mix)—was designed to probe the influence of the placement of two G:C base pairs within an otherwise repetitive A:T sequence and has been the subject of our prior experimental investigations using temperature-jump infrared spectroscopy and simple lattice models.^19^ We validate the new computational models of hybridization/dehybridization dynamics developed in this work against new experimental data and reinterpret our prior experimental observations in light of the new computational understanding. Consistent with previous studies,^18,25,30^ we find the degree of repetitiveness in the sequence—and therefore the kinetic accessibility and thermodynamic stability of out-of-register shifted states—leads to richer dynamics populated by a diversity of long-lived metastable states. Our data-driven modeling and analysis rigorously quantifies these behaviors and furnishes accurate predictive models of the hybridization/dehybridization rates, dynamical pathways, and metastable states. Specifically, we demonstrate that disrupting repetitive stretches of A:T bases by placement of interrupting G:C base pairs enables us to tune the landscape from rich six-state to simple two-state “all-or-nothing” behavior, and the specific location of the interrupting pair can be used to modulate the stability of long-lived frayed states. Taken together, our analyses establish new molecular-level understanding of the sequence-dependent kinetics and pathways through quantitative predictive models for the long-time system dynamics, resolution of the dynamical folding pathways and metastable states, and elementary design rules with which to control the dynamical behaviors of the system. We anticipate that this new foundational understanding, and the extension of our approach to more extensive families of DNA sequences, can guide the rational design of DNA oligomers with tailored kinetic properties engineered for DNA nanotechnology applications such as DNA-PAINT.^6,7^

## 2 Methods

### 2.1 Computational Methods

#### 2.1.1 Molecular Dynamics Simulations

We performed molecular dynamics simulations of four 10-base self-complementary double-stranded DNA sequences that we have previously studied by temperature-jump infrared spectroscopy:^19^ 5′-ATATATATAT-3′ (AT-all), 5′-GATATATATC-3′ (GC-end), 5′-ATATGCATAT-3′ (GC-core), and 5′-ATGATATCAT-3′ (GC-mix). We modeled the DNA sequences using the coarse-grained 3-Site-Per-Nucleotide v2 (3SPN.2) model that uses three spherical beads to represent the phosphate, deoxyribose sugar, and nitrogenous base of each nucleotide and employs anisotropic interaction potentials to accurately treat intrastrand base-stacking, interstrand cross-stacking, and base pairing.^49^ The model was parametrized against experimental data on bond lengths, bend angles, torsional angles, base step energies, and base stacking free energies, and reliably reproduces the structure, melting temperatures, persistence lengths, and sequence, salt, concentration, and temperature effects on duplex formation.^49^ The model enables access to millisecond time scales and has been has widely adopted to study numerous phenomena including DNA packing in viral capsids, protein–DNA binding, and nucleosome unwrapping.^69,77,78^ Although the 3SPN.2 model was not directly parametrized against dynamical experimental data, we will show below that the predicted sequence-dependent kinetics and relaxations are, within a corrective scaling factor, in good agreement with observed experimental trends.

All calculations were performed using the LAMMPS simulation package (http://lammps.sandia.gov) in accordance with best practices for the 3SPN.2 model.^79^ A single pair of self-complementary sequences were placed in a cubic periodic box with side length 7.8 nm corresponding to a single-strand concentration of 7 mM. This concentration is only 3.5× larger than the 2 mM concentration employed in our experimental analyses (section 2.2). Solvent effects were modeled implicitly by employing Langevin dynamics^80,81^ with an experimentally motivated per-site friction coefficient of 9.94 × 10^–11^ m^2^/s.^49,82^ We specified a 240 mM implicit salt concentration and treated electrostatic interactions using the Debye–Hückel with a 5 nm cutoff radius.^83^ Simulations were performed in the NVT ensemble employing a Langevin thermostat.^84^ With the exception of the simulation data reported in section 3.1 where we draw comparisons against experimental data collected over a range of temperatures, each sequence was simulated at its respective melting temperature rat 7 mM concentration as dictated by the 3SPN.2 model—AT-all: 309 K, GC-end: 317 K, GC-core: 324 K, GC-mix: 324 K—in order to maximize the number of spontaneous transitions between dissociated and hybridized states. Melting temperatures for each sequence were determined empirically by comparing the ratio of hybridized to dissociated populations over a 10 K temperature ramp centered on the nearest neighbor (NN) model predicted melting temperature,^8,9^ and selecting the temperature at which the hybridized and dissociated populations were approximately equal. The Langevin equations of motion were integrated using the scheme of Bussi and Parrinello^81^ with a 20 fs integration time step. We performed 40 independent simulations for each of the four sequences with half of the runs initialized from the hybridized state and half from the dissociated state. The initial hybridized state was defined based on the crystal structure coordinates of Arnott et al.^85^ The dissociated state was generated from the hybridized state by displacing one strand away from the other by 1 nm in each of the *x*, *y*, and *z* directions. The magnitude of this displacement did not affect the results so long as all native Watson–Crick (WC) bonds were completely broken. Initial bead velocities were assigned from a Maxwell–Boltzmann distribution at the temperature of interest. Each simulation was conducted for 26 μs and frames saved to disc every 100 ps. Each simulation required ∼24 CPU-hours on 28×Intel E5-2680v4 CPU cores. The first 1 μs of each run was discarded for equilibration providing us with 40 × 25 μs = 1 ms of simulation data for each sequence, during which time we observed 55–100 hybridization/dehybridization events.

#### 2.1.2 Markov State Model Construction

Markov state models (MSMs) are a powerful approach to infer long-time kinetic models from short molecular simulation trajectories^86−90^ that we employ in this work to construct high-resolution sequence-dependent kinetic models of DNA hybridization and dissociation. In brief, a MSM extracts from simulation trajectories an ensemble of long-lived metastable macrostates, their equilibrium occupancy populations, and the equilibrium probability fluxes between them. In this manner, they provide an interpretable and predictive model of the system thermodynamics and kinetics. MSMs have recently been implemented to study the hybridization mechanism of one particular 14-mer DNA oligomer, but determining the sequence-dependent kinetics and mechanisms was not the focus of this study.^91^ An energy disconnectivity graph-based approach was used to interrogate the differences in hybridization rates and mechanisms between GGGGGG and GCGCGC hexamers to reveal strong deviations from “all-or-nothing” behaviors and the importance of zippering and out-of-register diffusion mechanisms.^17^ Kinetic models were constructed not from the dynamical trajectories of the molecular model, but by estimating rate constants between local minima using a transition state theory approximation. A recent application of MSMs to the long-time dynamics of short RNA oligonucleotides revealed stacking time scales to be highly sequence dependent.^92^ In this work, MSMs were constructed for each of the four DNA sequences at their respective 3SPN.2 melting temperatures at 7 mM concentration from the 40 × 25 μs simulation trajectories following a four-step protocol detailed in ref (93): (i) trajectory featurization, (ii) dimensionality reduction, (iii) microstate clustering and microstate transition matrix inference, and (iv) macrostate clustering and macrostate transition matrix inference. Calculations were performed using the PyEMMA software package.^94^

##### Featurization

Trajectories comprising the Cartesian coordinates of the DNA strands as a function of time were featurized using the MDTraj Python libraries^95^ to represent the system in a manner that exposes the essential system dynamics but eliminates trivial translation and rotational invariances. We adopt intermolecular pairwise distances *d*(*i*,*j*) between the centers of mass of the 10 bases as a natural rototranslationally invariant featurization that represents each system configuration as the 10 × 10 = 100-element vector of interstrand pairwise distances. One additional symmetry arises from the self-complementary nature of these sequences—the sense and antisense strands in each pair are identical—such that the representation of the system under our featurization should remain unchanged upon inverting the arbitrary labeling of strand “1” and strand “2”.^86^ The 100-element pairwise distance vector is not invariant to this permutation, but can easily be made so via a simple symmetrization operation in which each of the  = 45 intermolecular pairwise distances are replaced by the mean of the two permutationally invariant distances. Specifically, (*d*(*i*_1_,*j*_2_) = *d*(*i*_2_,*j*_1_)) ← 0.5(*d*(*i*_1_,*j*_2_) + *d*(*i*_2_,*j*_1_)), where *i*_1_ denotes the *i*th base on strand 1 and *j*_2_ the *j*th base on strand 2.^86^ Finally, we took the reciprocal of the permutationally symmetrized pairwise distances to provide higher resolution and differentiation between proximate strand configurations in the near hybridized state compared to distantly separated dissociated strands. VAMP-2 scoring—calculation of the sum of the squared estimated eigenvalues of the transfer operator—of trajectories under a particular featurization provides a measure of the kinetic variance carried by that featurization.^96−99^ Performing VAMP-2 scoring at a lag time of τ = 1.2 ns and retaining the top five modes, reveals that the reciprocal permutationally symmetrized pairwise distances can carry up to twice the kinetic variance as the nonreciprocal distances, suggesting that the higher resolution offered at close intermolecular distances can indeed boost the dynamical representational power of the model. Somewhat surprisingly, we observed that augmenting our set of intermolecular distances between bases with intramolecular distances between bases on the same strand led to no improvement of the VAMP-2 score. This indicates that the kinetically relevant conformational state of the two strands are adequately represented via the intermolecular distances and leading us to employ only intermolecular distances within our featurization.

##### Dimensionality Reduction

The featurized trajectories were then projected into a low-dimensional space in preparation for microstate clustering. The standard approach to doing so is to employ time-lagged independent components analysis (tICA) to learn a linear projection into a low-dimensional embedding that maximally preserves the kinetic variance in the data.^97,100,101^ In this work, we instead employ state-free reversible VAMPnets (SRVs) that can be conceived of as a nonlinear version of tICA.^102^ SRVs employ neural networks to learn flexible nonlinear functions of the trajectory featurization that better approximate the slow dynamical modes of the system and have been shown to produce substantially higher resolution MSMs than those developed using tICA.^93,102^ SRV modes were learned independently for each system to best approximate the slow collective modes for that particular DNA sequence. SRVs were trained using the SRV package we previously developed (https://github.com/hsidky/srv) employing the default network architecture of two hidden layers each comprising 100 neurons and tanh activation functions, a learning rate of 0.001, and a batch size of 50 000. We adopted a lag time of τ = 1.2 ns as appropriately short time scale to resolve the dynamical details of the hybridization/dehybridization dynamics.^103^ As observed by Husic and Pande, the lag time cannot be treated as a hyperparameter to be optimized via the VAMP-2 score, but must be selected as a physically motivated choice designed to expose the dynamical motions relevant at a particular time and length scale of interest.^104^ As we shall show, this choice of lag time leads to high-resolution Markovian macrostate MSMs. We guarded against overfitting using 5-fold cross-validation in which we constructed five random partitions of the 40 independent simulation trajectories into a training set of 20 trajectories and a validation set of 20 trajectories.^93^ We observed plateau of the validation loss and no evidence of overfitting after 20 epochs of training requiring approximately 22 GPU-minutes on a single NVIDIA Tesla K80 GPU card. A VAMP-2 scoring of the cumulative kinetic variance explained as a function of number of SRV collective modes also showed no evidence of overfitting—as would be evinced by separation of the training and validation VAMP-2 scores^93^—and exhibited a knee for each of the four DNA sequences after the fifth, fourth, third, and second slow modes for AT-all, GC-end, GC-core, and GC-mix, respectively (Figure S1 in the Supporting Information), and motivating the construction of 5D, 4D, 3D, and 2D embeddings, respectively.

##### Microstate Clustering

The SRV projections of the 10 million frames recorded over the course of the 1 ms molecular simulation trajectories collected for each DNA sequence were then clustered into microstates using k-means clustering. The VAMP-2 score of the microstate transition matrix constructed for each sequence at the selected τ = 1.2 ns was insensitive to the choice of the number of microstates over the range 100–1000, motivating our selection of 200 microstate clusters for each system.

##### Macrostate Clustering

The 200 microstates comprising each system were coarsened into our terminal macrostate MSM. Since we are interested in the construction of models of the equilibrium kinetics, we explicitly enforce detailed balance in the construction of the MSM.^90^ The microstate transition matrix for each system was computed at a range of lag times τ and then diagonalized to recover the corresponding eigenvalues λ_*i*_ and associated implied time scales *t*_*i*_ = −τ/ln|λ_*i*_|.^90^ The implied time scale plots for the four DNA sequences are presented in Figure S2. We observe rapid convergence of the implied time scales *t*_*i*_ with lag time τ for all systems, motivating the construction of high resolution macrostate MSMs at a lag time τ = 1.2 ns. For this choice of lag time, we recover 5, 4, 2, and 1 implied time scales for the AT-all, GC-end, GC-core, and GC-mix systems, respectively. The identification of (*i* – 1) implied time scales implies the presence of (*i* – 1) slow modes and motivates the coarsening of the system into *i* macrostates. We estimate these *i* macrostates by applying PCCA+ spectral clustering to the leading (*i* – 1) eigenvectors of the microstate transition matrix.^105−107^ We then estimate the corresponding 6, 5, 3, and 2 macrostate transition matrices **P** for the AT-all, GC-end, GC-core, and GC-mix systems, respectively, by projecting the molecular simulation trajectories into these discrete macrostates. These macrostate MSMs constitute our terminal kinetic models. We validate the Markovian nature of the four MSMs by subjecting them to the Chapman–Kolmogorov (CK) test.^60,90,108^ This test asserts that the transition matrix for a Markovian (i.e., memoryless) MSM constructed at a lag time τ should satisfy the condition **P**(*k*τ) = **P**^*k*^(τ), which states that *k* successive applications of the transition matrix constructed at a lag time τ should be equivalent to a single application of the transition matrix constructed at a lag time *k*τ. We present in Figure S3 the CK tests for each DNA sequence to demonstrate that the τ = 1.2 ns models perform very well in predicting transition probabilities out to *k*τ = 7.2 ns, validating the Markovian nature and kinetic validity of the four models.

### 2.2 Experimental Methods

#### 2.2.1 Sample Preparation

Each DNA oligonucleotide was purchased from Integrated DNA Technologies (IDT) at desalt grade purity. Oligonucleotides were purified with 3 kDa cutoff centrifugal filters (Amicon). All labile protons were exchanged in deuterium oxide (D_2_O, Cambridge Isotopes, 99.9%). Oligonucleotides were prepared at a total strand concentration of 2 mM in 50 mM pD 7.2 sodium phosphate buffer with 240 mM NaCl and 18 mM MgCl_2_. Prior to each measurement, DNA solutions were placed in a water bath at 90 °C for 3 min and then cooled to room temperature under ambient conditions.

#### 2.2.2 T-Jump IR Spectroscopy

The details of the technique and processing used to acquire temperature-jump infrared (T-jump IR) data have been described previously.^109−111^ Briefly, heating was initiated through optical excitation of the O–D stretch overtone transition of D_2_O. The 1.98 μm pulses (5 ns, 20 mJ, 20 Hz) used for heating were generated from the frequency-doubled output of a Nd:YAG laser sent through an optical parametric oscillator. Nonlinear IR spectra are collected from 5 ns to 50 ms delays after the T-jump with a synchronized 1 kHz spectrometer. T-jump heterodyne-detected vibrational echo (t-HDVE) IR spectra were acquired with Fourier transform spectral interferometry,^110^ where the delay between the local oscillator (LO) and DVE signal was scanned in 5 fs steps between (−10) and 10 fs. t-HDVE spectra were acquired with a parallel pulse polarization scheme and presented as a dispersed pump–probe (t-DPP) spectrum. t-DPP data are reported as the difference spectra relative to the initial temperature.

The sample was placed between two 1 mm thick CaF_2_ windows separated by a 50 μm Teflon spacer enclosed in a brass jacket. The initial temperature of the sample was set using a recirculating chiller connected to the brass sample jacket. The T-jump temperature change (Δ*T*) was set to 14–16 °C for all measurements and monitored using the change in transmission of the D_2_O bend–libration combination band measured in the LO beam. The temperature change was quantified by comparing the change in transmission of the LO beam with a FTIR temperature series of D_2_O.

#### 2.2.3 Determination of Fast and Slow Dissociation Rates

To determine observed rates from the T-jump data, the time-domain t-HDVE data was inverse Laplace transformed into the rate domain using a maximum entropy approach (MEM-iLT).^112^ Observed rates λ^fast^ and λ^slow^ were computed from the amplitude-weighted mean rate across detected IR frequency, as previously described.^28^ The fast response *k*_d_^fast^ is defined as this amplitude-weighted mean rate, whereas the dissociation rate constant *k*_d_^slow^ was extracted from the observed rate of the slow response λ^slow^ using a two-state model for self-complementary oligomers,^113^
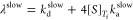
1where [*S*]_*T*_f__ is the concentration of single-strand oligomer at the final temperature of the T-jump, and *k*_a_^slow^ is the association rate constant. In practice, eq 1 is recast in terms of the dissociation equilibrium constant *K*_d_ to solve for *k*_d_^slow^ and *k*_a_^slow^ as a function of *K*_d_ and [*S*]_*T*_f__,
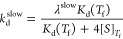
2
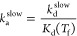
3

FTIR temperature series were measured for each sequence as reported previously,^19^ and the second SVD component along temperature was fit to a two-state model to determine the fraction of intact duplex θ as a function of temperature.^114^*K*_d_ and [*S*]_*T*_f__ were then determined from θ at the final temperature of each T-jump measurement,
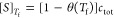
4
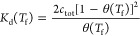
5where *c*_tot_ is the total oligonucleotide concentration, which was 2 mM for all measurements.

## 3 Results and Discussion

### 3.1 Sequence-Dependent Coarse-Grained Kinetics Recapitulate T-Jump IR Measurements

We first sought to demonstrate that the coarse-grained 3SPN.2 model accurately recapitulates the experimentally observed kinetics of DNA oligomer hybridization/dissociation by validating our computational predictions against temperature-jump infrared (T-jump IR) experiments. We conducted T-jump IR experiments for each DNA sequence as a function of temperature and extracted the “slow” *k*_d_^slow^ and “fast” *k*_d_^fast^ rates, corresponding to processes proceeding on 10–30 μs and 70–100 ns time scales, respectively. The slow response has previously been attributed to duplex dissociation on microsecond time scales induced by the rapid heating of the initially hybridized duplex.^19,28^ The fast response has been assigned to terminal base pair fraying,^19,28^ with this process corresponding to a relatively complex dynamical process that can span time scales from picoseconds to microseconds.^33−35,115^ All-atom simulations suggest that frayed ends can assume misaligned WC bonds, base-sugar hydrogen bonds, and terminal stacked conformations.^32,116^

To computationally mimic the T-jump process in our 3SPN.2 simulations, we conducted 1 μs simulations of an initially hybridized DNA duplex over a range of temperatures and monitored its structural relaxation. We performed 120 independent simulations for each DNA sequence at each temperature, and from these extracted computational estimates of *k*_d_^slow^ and *k*_d_^fast^ (Figure S4). First, we tracked the slow response corresponding to duplex dissociation in our simulations by compiling the distribution of times at which both of the central base pairs first separate to a distance of 2.0 nm starting from an initial fully hybridized duplex. This cutoff was selected as the distance beyond which the strands are effectively non-interacting and defines the dissociated state. We extracted our computational estimate of *k*_d_^slow^ by fitting a decaying exponential to the fraction of hybridized sequences as a function of time *f*_hybridized_(*t*) = exp(−*k*_d_^slow^*t*). We verified that our cutoff was sufficiently large by observing that our calculated values for *k*_d_^slow^ changed by an average of only 7% by adopting a 1.3 nm cutoff.

Second, we tracked the fast response corresponding to terminal base pair fraying by compiling the distribution of times at which either of the terminal base pairs first separated to a distance of 1.3 nm, corresponding to a complete breakage of the WC interaction. We extracted our computational estimate of *k*_d_^fast^ through a decaying exponential fit to the fraction of unfrayed sequences as a function of time *f*_unfrayed_(*t*) = exp(−*k*_d_^fast^*t*).

**Figure 1:**
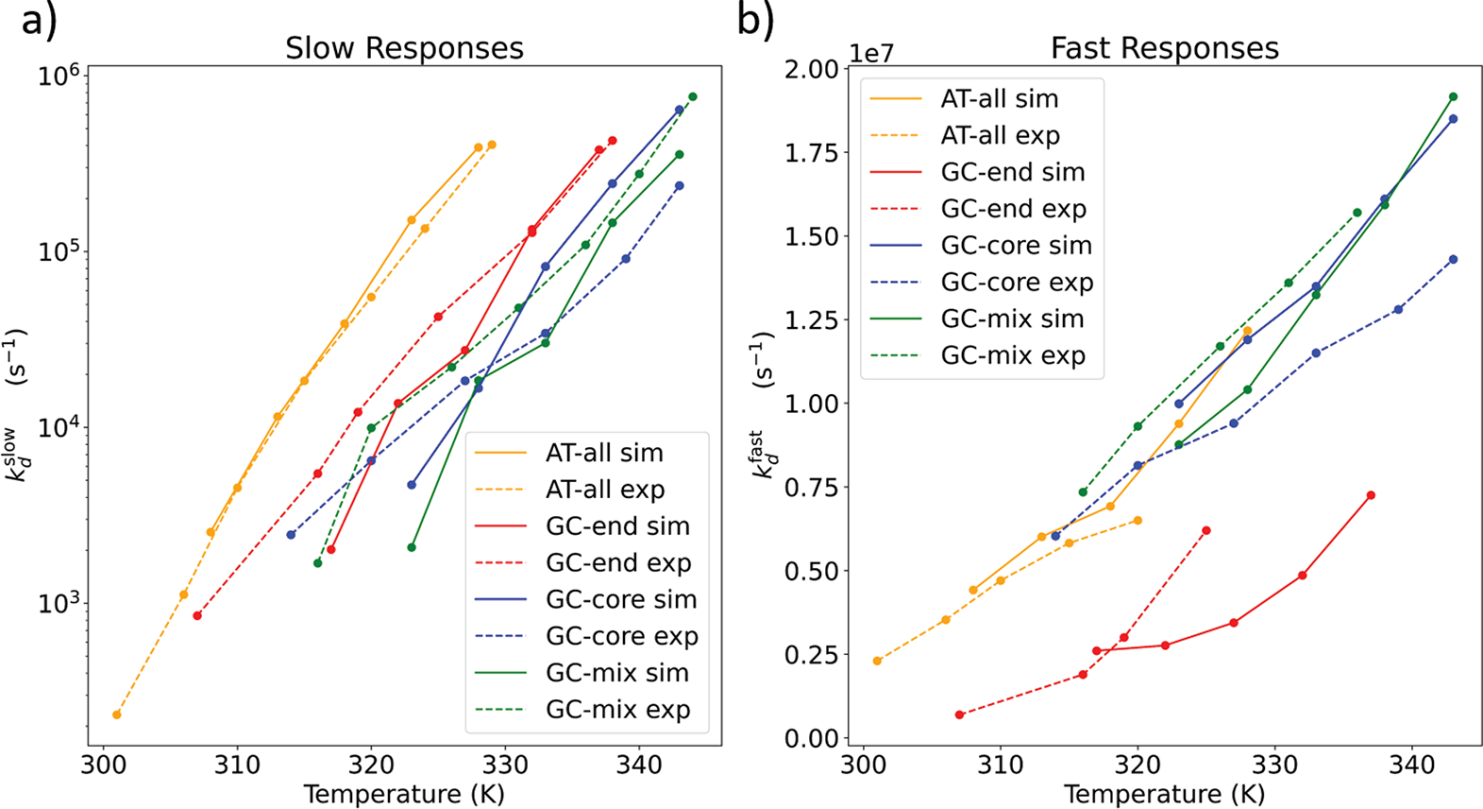
Experimental measurements and computational predictions of slow and fast at T-jump IR responses. Results are reported in terms of the final T-jump temperature. (a) The experimental and simulated slow rate constants *k*_d_^slow^ corresponding to duplex dissociation over long time scales. (b) The experimental and simulated fast rate constant *k*_d_^fast^ corresponding to terminal base-pair fraying on short time scales. The simulation results are corrected by a sequence-independent scaling factor that corrects for a 10× acceleration of the slow dissociation dynamics and 120× acceleration of the fast fraying dynamics. The simulated temperature in all cases is subjected to a (+4) K corrective calibration to account for an observed systematic under-prediction of the melting temperature by the 3SPN.2 model

We present in Figure 1 a comparison of *k*_d_^slow^ and *k*_d_^fast^ estimated by computation and experiment. Although the 3SPN.2 model was not directly fitted against kinetic data,^49^ its predictions of sequence-dependent T-jump relaxation rates are, within a systematic scaling factor in time and systematic shift in temperature, in good agreement with observed experimental trends. It is well-known that the smoothing of the underlying free energy landscape induced by coarse-graining artificially accelerates the kinetics of coarse-grained molecular simulations and that different degrees of freedom may be accelerated by different factors.^117−119^ We find that the simulated slow responses corresponding to center-of-mass translation of the strands during dissociation of the duplex is ∼10× accelerated relative to experiment, whereas the fast responses corresponding to fraying of the terminal bases is ∼120× accelerated. We apply these sequence-independent scaling factors to our reported computational values in Figure 1. Although the 3SPN.2 model reproduces melting temperatures relatively well, we observed a systematic 4 K under-prediction relative to experiment and so we apply a universal (+4) K corrective temperature shift to our computational results. We note that our simulations are expected to slightly over-predict the melting temperature since they are conducted at 3.5× higher concentration relative to experiment, and the concentration-adjusted corrective shift accounting for the approximations inherent in the 3SPN.2 model would be slightly larger. Owczarzy et al. present an analytical prescription to apply concentration corrections to the melting temperature using knowledge of the enthalpy changes associated with duplex formation and helix nucleation.^120^ In the absence of these quantities, one could instead empirically estimate concentration corrected melting temperatures by conducting a suite of simulations over a range of temperatures and assume ideal molecular behavior to apply concentration corrections to the observed hybridized fractions.^121^ (As demonstrated by Ouldridge et al., finite-size corrections do not affect the melting temperature for homodimers.^121^) Furthermore, we note that these empirical calibration factors to the 3SPN.2 predictions are applied only for the purposes of making an experimental comparison, but acknowledge that there are uncertainties introduced by assuming that the equilibrium dynamics at fixed temperature can be compared directly to relaxation kinetics following a 15 °C T-jump. The computational time scales and melting temperatures reported in the remainder of the paper are not corrected by these calibration corrections since we are only interested in the relative trends in the behaviors of the four sequences.

In Figure 1a we observe an exponential increase of *k*_d_^slow^ with temperature, as expected from the large enthalpic barrier to duplex dissociation.^20,38,40^ Under the time and temperature calibration corrections, we see generally very good agreement between the computational and experimental curves. Of the four sequences, GC-core shows the largest discrepancy between computation and experiment, although the general exponential trend is preserved. This may be a result of its high propensity to fray (cf. section 3.5). In Figure 1b we expose a largely linear dependence of *k*_d_^fast^ upon temperature for AT-all, GC-end, and GC-mix compared to an exponential dependence for GC-end. These trends can be understood in light of the comparatively larger enthalpic barrier for dissociation of the terminal G:C base pair in GC-end compared to that for the A:T terminal pair in the other three sequences. Again we see good agreement between the scaled computational predictions and the experimental T-jump measurements. The favorable comparison of computation and experiment provides support for the capacity of the 3SPN.2 model to reliably reproduce sequence-dependent trends in the slow and fast kinetics of the four DNA oligomers, and lends confidence in the use of these data for the parametrization of Markov state models of the long-time dynamics of each sequence. We observe that the dynamical fingerprinting approach presents an elegant means to compare experimental relaxation data directly against a Markov state model extracted from simulation data in terms of a “fingerprint” of peaks with amplitudes and time scales related to the relaxation of particular system observables.^122^ These techniques have been previously employed to in applications to base stacking of DNA dinucleoside monophosphates^123^ and RNA.^92^ We explored the use of this approach to validate the MD simulation data but encountered challenges in resolving fast dynamical motions below the lag time of the fitted MSM and the extremely high computational cost of collecting sufficient simulation data to fit MSMs at each temperature for which experimental data was collected. Accordingly, we instead elected to perform a direct comparison between the experimental data and MD trajectories to validate the simulations themselves, then proceed to train MSMs over these data and conduct analysis and experimental tests of the MSM predictions.

### 3.2 SRV-MSMs for Each Sequence

**Figure 2:**
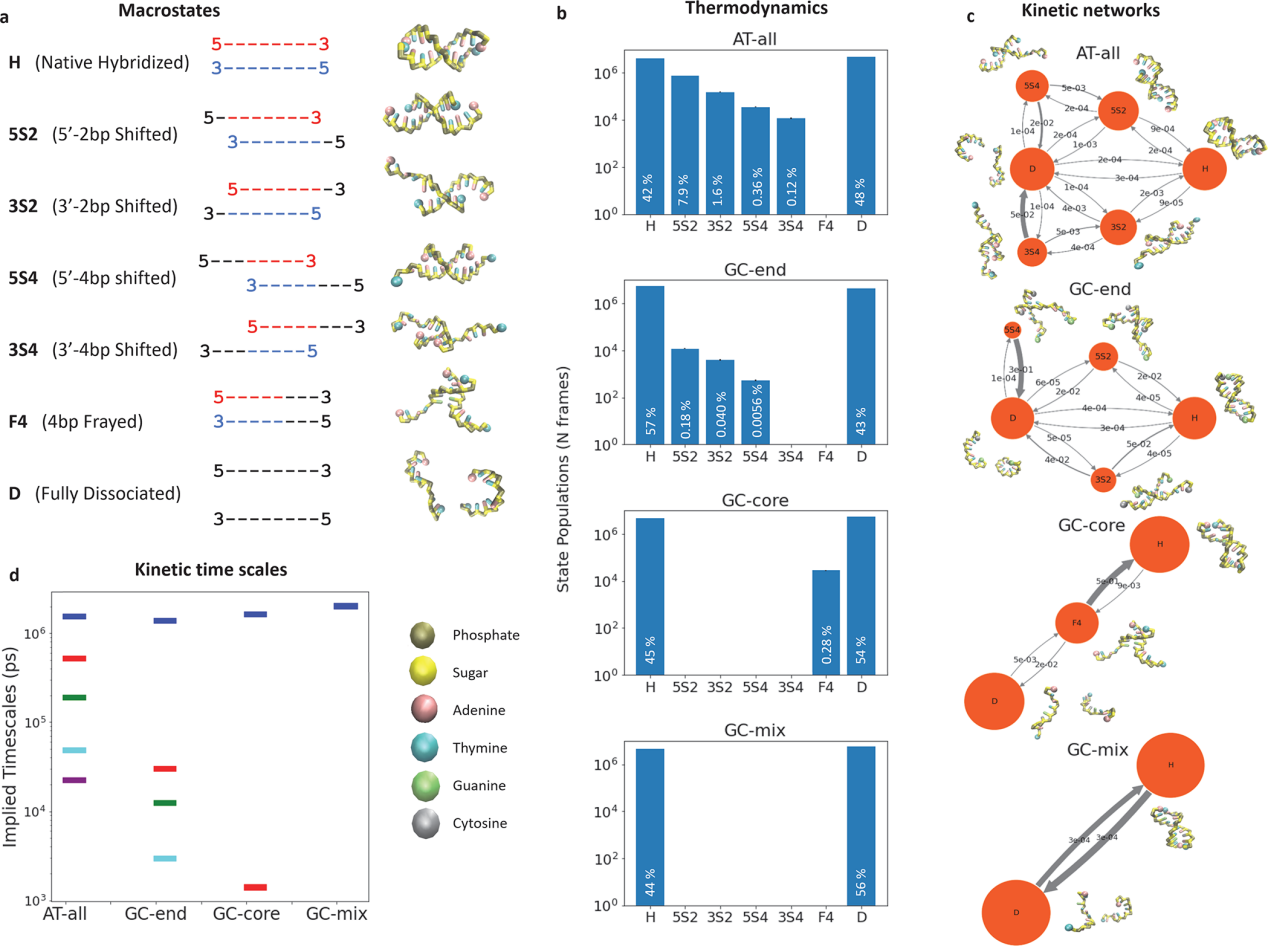
Thermodynamic and kinetic predictions of the sequence-specific MSMs fitted at the sequence melting temperatures AT-all, 309 K; GC-end, 317 K; GC-core, 324 K; and GC-mix, 324 K. (a) Macrostates: Schematic representation of the seven metastable macrostates occupied by one or more of the four sequences: fully hybridized state (H), two-base out-of-register shifted states in the 5′ (5S2) or 3′ (3S2) direction, four-base out-of-register shifted states in the 5′ (5S4) or 3′ (3S4) direction, and the fully dissociated state (D). The line drawings represent the 10-base self-complementary sequences, where red-to-blue contacts indicates (possible) WC base pairing and black indicates an unbound bases. Adjacent to each line drawing we provide representative molecular structures corresponding to that macrostate. (b) Thermodynamics: Histograms reporting the number of the 10^7^ total frames within the 1 ms of simulation trajectories observed to occupy each of the seven macrostates, corresponding to our numerical estimates of the equilibrium occupancy probabilities. Uncertainties are calculated across 100 MSMs using a Bayesian MSM estimation are reported for each bar and are very small compared to the total counts. Values are reported on a log *y*-axis to make the small populations of the shifted and frayed states visible. (c) Kinetic networks: MSMs illustrating the kinetic network for each sequence. The orange circles correspond to the macrostates occupied by each sequence and are labeled by the macrostate codes reported in panel a. The area of the circles is proportional to the logarithm of the equilibrium occupancy populations reported in panel b. Molecular renderings of an illustrative snapshot from the coarse-grained molecular simulations are provided next to each macrostate. The gray arrows between macrostates indicate the presence of a probability flux between this pair of states at equilibrium and the arrow thickness is proportional to the flux. (To avoid congesting the diagram, arrows are not reported for probability fluxes lower than 3 × 10^–6^.) The numerical value overlaid on each arrow reports the conditional probability that a system occupying the macrostate at the start of the arrow at time *t* will transition to the macrostate at the end of the arrow by time (*t* + τ), where τ = 1.2 ns is the lag time corresponding to a single time step of the MSM. Large orange circles correspond to thermodynamically favorable states and large gray arrows correspond to kinetically favorable transitions. (d) Kinetic time scales. Distribution of MSM implied time scales for each sequence. The leading implied time scale corresponds to the characteristic time scale for hybridization/dehybridization and is approximately equal for all systems since simulations were conducted at the same concentration and at the respective melting temperatures. The higher order implied time scales correspond to a spectrum of kinetic relaxations between the constituent macrostates in the MSM corresponding to shifted and/or frayed states.

We then proceeded to construct Markov state models (MSMs) from 1 ms of aggregated simulation trajectories for each of the four sequences at their respective melting temperatures to generate sequence-dependent kinetic models. MSMs define the long-lived metastable macrostates of the system, their equilibrium occupancies, and the equilibrium transition probabilities between them. As such, they are extremely valuable in providing both a quantitative predictive model and a physically comprehensible mechanistic understanding of the long-time dynamical evolution of the system between an ensemble of metastable macrostates. We present in Figure 2 the inferred MSMs for each of the four 10-base DNA sequences. Across all four sequences we identify a totality of seven metastable macrostates corresponding to the fully hybridized state (H) in which all native base pairings are intact, four shifted states in which the strands are translated out-of-register by two or four bases in the 5′ (5S2, 5S4) or 3′ (3S2, 3S4) direction, a frayed state (F4)—unique to GC-core—in which four terminal A:T base pairs are unbound, and the fully dissociated state (D). We present these seven macrostates in Figure 2a along with schematic and cartoon renderings of representative microstates contained within each of these macrostates. The representative microstates emblematic of each macrostate were selected randomly from those possessing high (>99%) macrostate membership probabilities. Since the microstate ensemble exhibits a range of conformations, however, it is useful to characterize the degree of structural heterogeneity to determine the degree to which these microstates are emblematic of the distribution. In Figure S5 we present a projection of our macrostates into two physical order parameters—the degree of 3′ shift and degree of 5′ shift—to provide an interpretable embedding of the macrostates that exposes base pairing patterns and structural heterogeneity within the microstate ensemble. In all cases, we find these microstate distributions to be relatively narrowly focused within the low-free energy core of each macrostate such that a single archetypal microstate is indeed a good representative for the ensemble and an accurate representation of the base pairing pattern of the macrostate. In Figure 2b we present the occupancy probabilities of each state at thermodynamic equilibrium. By virtue of the fact that each sequence is simulated at its corresponding melting temperature (*T*_m_), the probability of the dissociated state (*D*) is, by construction, approximately equal to the sum of the probabilities over the remaining six states (H, 5S2, 3S2, 5S4, 3S4, F4). In Figure 2c we present a visualization of the macrostate MSMs for each of the four sequences showing the connectivity between the identified macrostates. The macrostates are represented as orange circles in proportion to their equilibrium probabilities and the gray arrows indicate the probability of hopping from one macrostate to another under one time step of the kinetic model. The fluxes between the macrostates provide a wealth of high-level, interpretable information on the sequence-dependent metastable states and hybridization/dehybridization pathways. Immediately, we identify that the AT-all sequence possesses a rich and complex dynamical landscape comprising six metastable states whereas at the other end of the spectrum GC-mix exhibits far simpler two-state “all-or-nothing” behavior. In Figure 2d we present the so-called implied time scales of each MSM. These time scales correspond to the relaxation times of the DNA dimer among its constituent metastable macrostates. The leading implied time scale for each system corresponds to the characteristic time scale for hybridization/dehybridization. Since each system is simulated at the same 7 mM concentration and at its respective melting temperature, it is not surprising that the leading time scale is approximately equal for all four systems and corresponds to the characteristic time scale for hybridization/dehybridization. The spectrum of higher order time scales corresponds to increasingly quicker relaxations between the metastable macrostates within the kinetic model and resolve the interesting sequence-dependent differences in the hybridization/dehybridization kinetic pathways. The total number of implied time scales is typically one fewer than the number of metastable macrostates and the existence of large implied time scales is indicative of slowly relaxing kinetic processes. The dense spectrum of slow implied time scales for AT-all is indicative of its relatively complex kinetic landscape whereas that for the two-state GC-mix comprises only a single time scale corresponding to hybridization/dehybridization. We now proceed to analyze in detail the sequence-dependent thermodynamics, kinetic, and mechanisms exposed by the four MSMs.

### 3.3 Comparison of MSM Thermodynamic Predictions with NN Model

We first compare the thermodynamic predictions for the equilibrium macrostate probabilities made by the MSM models fitted at the sequence 3SPN.2 melting temperatures to those of the nearest neighbor (NN) model as a popular empirical model of DNA hybridization thermodynamics. The NN model predicts the free energy of duplex formation as a sum over helix initiation terms and the hybridization free energies of nearest neighbor pairs of bases that account for both the specific WC pairings and the modulating effects of the local (i.e., nearest neighbor) environment.^8,9^ The parameters of the NN model were estimated by regressing over 108 experimental measurements to furnish a predictive model for the free energy of association as a function of DNA sequence and explicitly account for stacking contributions of native pairs, internal mismatches, and dangling ends. We apply the NN model to predict the free energy *F*^NN^ of each of the macrostates occupied by each of the four sequences. A full accounting of our application of the NN model is provided in Figure S6 and supporting text within the Supporting Information. The free energy of each macrostate is related to its equilibrium occupancy probability *P* via the statistical mechanical relationship *F* = −*k*_B_*T* ln *P* + *C*, where *T* is temperature, *k*_B_ is Boltzmann’s constant, and *C* is an additive constant reflecting our ignorance of the absolute scale of free energies. We use this relationship to convert the equilibrium occupancy probabilities predicted by our MSM and reported in Figure 2b into free energies *F*^MSM^. The unknown additive constants preclude comparisons of absolute free energies between the MSM and NN model, but it is legitimate to compare relative free energies between pairs of macrostates since the additive constant cancels in taking differences. As such, we arbitrarily set the additive constant *C* in both the MSM and NN model such that the hybridized state H defines a zero free energy reference state and we report the stability of all macrostates relative to the hybridized state as Δ*F*^NN^ = *F*^NN^ – *F*_H_^NN^ for the NN model and Δ*F*^MSM^ = *F*^MSM^ – *F*_H_^MSM^ for the MSM.

**Figure 3:**
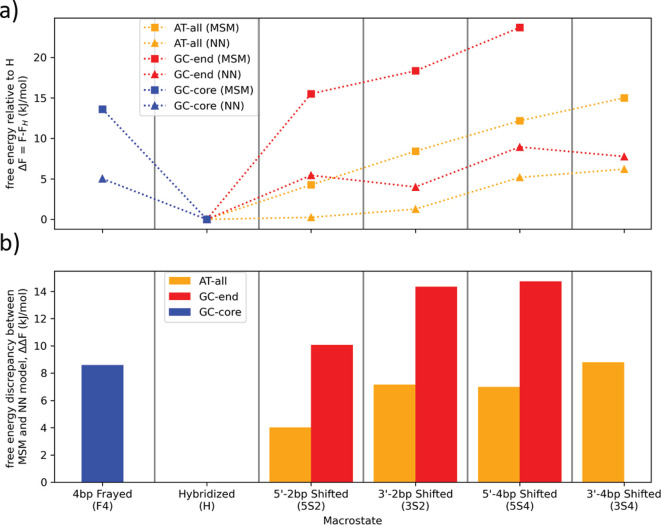
Comparison of the macrostate free energy predictions of the MSMs and nearest neighbor (NN) thermodynamic model at the sequence 3SPN.2 melting temperatures.^8,9^ (a) Free energies of each macrostate relative to the hybridized state Δ*F* = *F* – *F*_H_. We define the hybridized state H to possess a free energy of zero and take care to only compare relative free energies (i.e., Δ*F*) between the MSM and NN model. (b) Discrepancy between the macrostate relative free energy predictions ΔΔ*F* = Δ*F*^MSM^ – Δ*F*^NN^ of the MSM relative to the NN model. The MSM tends to predict higher relative free energies (i.e., lower occupancy probabilities) relative to the hybridized state H compared to the NN model.

As illustrated in Figure 3, we see that the MSM tends to predict higher free energies for all macrostates relative to the H state compared to the NN model. Although the trends are in qualitative agreement, what is the root of the quantitative discrepancy of the MSM and NN models in the predicted relative stabilities? First, the MSM is constructed bottom-up from molecularly detailed 3SPN.2 simulations whereas the NN model is fitted top-down by regression against experimental data. There are approximations inherent in the 3SPN.2 model, not least of which is the coarse-grained representation that integrates over atomic degrees of freedom, and in the NN model that was fitted to limited experimental data assuming a low-order expansion in terms of nearest neighbor additive contributions. Second, although 3SPN.2 is expected to capture some dangling end stabilization effects through base stacking and cross-stacking interactions, the model was not parametrized to fully capture the enthalpic contributions of the interaction of unbound bases with terminal base pairs, whereas this term is explicitly included within the NN model. Third, a well-known deficiency of the NN model is the absence of any treatment of inert tails—free bases that extend beyond the dangling end tend to destabilize the duplex.^124^

We can further explore the role of inert tails upon macrostate stability by analyzing the AT-all and GC-end sequences that occupy out-of-register shifted macrostates 5S2 and 3S2 comprising a one-base inert tail and 5S4 and 3S4 comprising a three-base inert tail (cf. Figure 2a). For both sequences, our simulations show that 5S2 is both the most stable of shifted states relative to H (Figure 3a) and has the smallest discrepancy (∼4 kJ/mol AT-all, ∼10 kJ/mol GC-end) compared to NN predictions (Figure 3b). The 3S2 and 5S4 states have nearly the same deviation from NN predictions (∼7 kJ/mol AT-all, ∼ 15 kJ/mol GC-end), indicating that longer tails in the 5′ direction are as destabilizing as shorter tails in the 3′ direction. It is known from differential scanning calorimetry studies^125^ that 3′ inert tails are more destabilizing than 5′ tails, with the differential behavior attributed to a combination of 5′ tails preferentially stacking on the core duplex and 3′ tails perturbing the duplex structure.^124−126^ Both the NN and MSM predictions for AT-all are consistent with this trend (i.e., *F*_3S2_ > *F*_5S2_ and *F*_3S4_ > *F*_5S4_). For GC-end, the MSM and NN models both predict the out-of-register shifted states to be less stable relative to the hybridized state than the corresponding predictions for AT-all. This is in line with expectations since the terminal G:C pairs in GC-end decrease by two the number of available WC pairings in out-of-register shifted states compared to AT-all. The GC-end NN predictions run contrary to the expectation that the 3′ inert tails should be more destabilizing than the 5′ tails, whereas the MSM predictions are consistent with this trend. Indeed, the MSM model for GC-end does not identify the 3S4 macrostate as a metastable conformation for the duplex.

In sum, the qualitative trends in the macrostate thermodynamic stabilities are in good agreement between the MSM and NN models, but show quantitative discrepancies for macrostates possessing inert tails. In these instances the MSM predicts these macrostates to be less stable relative to the hybridized state compared to the NN model predictions by 4.0–14.5 kJ/mol. The MSM predictions are also consistent with the experimental expectation that 3′ inert tails should be more destabilizing than the 5′ tails, whereas the NN predictions can be in conflict with this trend.

### 3.4 Out-of-Register States Facilitate Hybridization and Dissociation Dynamics (AT-All, GC-End)

In addition to thermodynamic stabilities, the macrostate MSM also furnishes quantitative and interpretable predictions of hybridization and dehybridization pathways and mechanisms at the sequence melting temperatures. It should be noted that because we are using a reversible MSM framework, detailed balance is enforced by construction. We now proceed to analyze these predictions for each of the four sequences and illuminate the relationship between sequence and dynamics. Two of our sequences, AT-all and GC-end, support out-of-register metastable states, and we commence our analysis with the role of these shifted states.

AT-all possesses the richest and most complex MSM of the four sequences by virtue of its repetitive nature, comprising a hybridized state (H), dissociated state (D), and four out-of-register shifted states (5S2, 3S2, 5S4, 3S4) (Figure 2c). Analysis of the MSM transition probabilities reveal a critical role of the shifted states in mediating hybridization and dehybridization. Commencing from the dissociated state D, we observe approximately equal probabilities for transitions to each of the other five states, such that a transition to one of the out-of-register shifted states 5S2, 3S2, 5S4, or 3S4 is approximately 2.2 times more likely than a direct transition to the hybridized state H. Commencing from the hybridized state H, however, a direct transition to the dissociated state is approximately 1.2 times more likely than a transition to one of the two-base shifted states 5S2 or 3S2. Once in one of the four shifted states, the 5′ vs 3′ overhang and degree of shifting play an important role in determining whether the duplex will transition to more shifted states, more aligned states, or completely dissociate. Transitions from more shifted states toward more aligned states (i.e., 5S4 → 5S2, 5S2 → H, 3S4 → 3S2, 3S2 → H) are approximately an order of magnitude more probable than the reverse transitions from more aligned states to more shifted states. The largest single transition probability from the four shifted states 5S2, 3S2, 5S4, and 3S4 is, however, back to the dissociated state D. Consistent with the higher destabilizing effect of 3′ inert tails relative to 5′ tails,^124−126^ the 3S4 → D transition probability is twice as large as the 5S4 → D, and the 3S2 → D is four times larger than the 5S2 → D. The transition probability from the 5S2 and 3S2 states back to the dissociated state D is equal to or greater than the transition probability to the hybridized state. A transition path theory analysis of the MSM reveals that 33% of productive hybridization trajectories D --> H (where the dashed arrow indicates the combination of both direct and indirect pathways) and dehybridization trajectories H --> D proceed through one or more out-of-register shifted states. Among these out-of-register hybridization pathways, the D --> 5S2 --> H transition is predicted to occur 57% of the time. A mean first passage time (MFPT) analysis returns a MFPT for D --> H of 3.0 μs and for H --> D of 2.5 μs. As expected by the fact that the calculations are performed at the melting temperature, the MFPTs are approximately equal.

GC-end comprises the next most complex MSM. The introduction of the G:C pairs at the termini of the strands maximally preserves the repetitive tract of A:T base pairings such that the GC-end MSM possesses all of the same macrostates in its dynamical landscape with the exception of the 3S4 state (Figure 2c). As discussed in section 3.3, the 3S4 state is rendered unstable within the lag time of our MSM due to the presence of the destabilizing 3′ inert tail and only four WC base pairings compared to six in the case of AT-all. Analysis of the transition probabilities reveal significant differences compared to those in the AT-all kinetic network. Commencing from the dissociated state D, we observe a similar transition probability to the 5S4 state as for AT-all, but once in the 5S4 state there are no significant transition probabilities to any other state except back to D. As such, the 5S4 state acts as a kinetic trap rather than as an intermediate to hybridization. The D → H and H → D transition probabilities are commensurate with those for AT-all. However, the D → 5S2 and D → 3S2 transition probabilities are half or less of those in AT-all, and the reverse transitions are an order of magnitude larger. This may be attributed to the reduced thermodynamic stability of the 5S2 and 3S2 states in GC-end that comprise only six WC pairs compared to eight in AT-all (cf. Figure 3). The 5S2 → H and 3S2 → H transition probabilities are more than an order of magnitude larger than in AT-all, which may again be attributed to the lower thermodynamic stability of the two shifted states relative to the hybridized state H. Again, the transition probabilities out of the 3S2 state to D or H are comparatively higher than those out of the 5S2 state, consistent with the increased destabilizing effect of 3′ inert tails.^124−126^ Commencing from the hybridized state H, a direct transition to the dissociated state is approximately seven times more likely than a transition to one of the two-base shifted states 5S2 or 3S2. A transition path theory analysis of the MSM reveals that only 7% of hybridization events D --> H and dehybridization events H --> D proceed through one or more out-of-register shifted states. The significantly reduced role for out-of-register shifted states in mediating the hybridization and dissociation pathways for GC-end relative to AT-all is consistent with the reduced thermodynamic stability of these states due to the elimination of possible out-of-register WC base pairing for the terminal G:C pairs and therefore a reduced accessibility of these states in the GC-end kinetic network. We compute a MFPT for D --> H of 1.6 μs and for H --> D of 2.1 μs, which are again approximately equal.

The out-of-register kinetic landscape that defines AT-all and GC-end hybridization have been explored by a number of previous computational studies. Simulations have identified internal displacement mechanisms capable of correcting base pair alignment in 3SPN.2^18^ as well as in the coarse-grained oxDNA^30^ and BioModi^67^ models. In all cases, these mechanisms were shown to be crucial components of the hybridization pathway for homogeneous and repetitive sequences. Xiao et al. performed an all-atom energy landscape-based analysis of 5′-GGGGGG-3′ and 5′-GCGCGC-3′ hexamers.^17^ Out-of-register states for 5′-GCGCGC-3′ hexamers were identified as deep kinetic traps along the hybridization pathway and “slithering” through these states did not provide a significant hybridization pathway compared to an alternative “zippering” mechanism. (In contrast, slithering through out-of-register shifted states and zippering served as two parallel pathways for hybridization of 5′-GGGGGG-3′.) This stands in contrast to our results for our AT-all (5′-ATATATATAT-3′) sequence, in which out-of-register states participated in 33% of productive hybridization events. It is conceivable that the stronger hydrogen bonding in G:C WC pairs relative to A:T pairs may render out-of-register shifted states less favorable to hybridization by suppressing fluctuation-driven rearrangements,^127,128^ but additional studies would be required to reconcile these observations.

### 3.5 Central GC Placement Induces Long-Lived Frayed States (GC-Core)

The GC-core MSM represents a departure from the relatively rich and complex kinetic networks dominated by out-of-register shifted states to a much simpler one dominated by fraying (Figure 2c). The MSM contains only three states—hybridized H, dehybridized D, and frayed F4. The F4 state is unique to GC-core and contains up to six WC pairs—the two central G:C core pairs and as many as four A:T pairs on one side or other of the core, while the other run of four A:T pairs remains free. (As expected by symmetry, the particular AT run that is free occurs with equal probability on either side of the core.) Although partially frayed states containing less than four free A:T bases on either end of the duplex are common, these tend to interconvert faster than the lag time and are not registered as metastable within our MSM. Our model reveals the absence of any direct hybridization or dehybridization transitions between the H and D states, with all pathways passing through the frayed state F4. Previous studies would suggest that hybridization of this sequence should proceed via a zippering mechanism, wherein upon formation of the strong central G:C WC base pairings the duplex helix should rapidly assemble in a middle-out fashion.^16,30^ Our results are partially consistent with this expectation, but reveal the frayed state F4 to be unexpectedly metastable, serving as a long-lived state with an mean lifetime of 1.8 ns. The stability of the state is attributable to the enthalpic stabilization offered from up to six WC pairs and the entropic stabilization associated with the configurational entropy of the two free AT-tails.

Analysis of the transition probabilities show that commencing from the F4 state, progression to the hybridized state F4 → H is 25 times more likely than dissociation F4 → D. Thus, once a D → F4 transition has occurred, a F4 → H transition will likely proceed; concomitantly, H → F4 events tend to fall back to the H state and are less likely to proceed to complete dissociation D. We noted in section 3.1 that fraying dynamics in the 3SPN.2 model appear to be significantly accelerated relative to center-of-mass translation, and it is conceivable that this may lead to elevated sampling of the F4 state within the computational model relative to experiment and the induction of more frequent dissociation. Moreover, since GC-core is the sequence most prone to fraying, this effect could be the root of the relatively poorer agreement of the *k*_d_^slow^ response for GC-core compared to the other sequences due to an artificially elevated computational prediction of this rate (Figure 1a). Our model predicts a MFPT for D → F4 → H of 3.4 μs and for H → F4 → D of 2.9 μs, which are again approximately equal.

Lattice models have previously identified frayed states as putative intermediates in DNA hybridization/dehybridization.^19,25,46^ Araque et al. studied a 5′-ATGCGCAT-3′ octomer using a lattice model and identified a symmetrically A:T frayed state as a crucial part of the duplex transition path.^25^ We previously studied the four sequences that are the subject of the present work using T-jump IR and 2D IR spectroscopy and identified GC-core as possessing the highest deviation from two-state behavior during dissociation when neglecting out-of-register contributions.^19^ This result was interpreted to arise from loss of A:T contacts and fraying around the central G:C core, and this hypothesis was supported by lattice model calculations that predicted the GC-core conformational ensemble to possess substantially more frayed configurations than the other three sequences.^46^ Follow-up T-jump measurements and Smoluchowski simulations on model 1D free energy landscapes showed that AT termini fraying was an effectively barrierless process characterized by rapid interconversion between all accessible frayed states.^28^ These prior results are consistent with the present findings that expose the GC-core sequence to be the only sequence that occupies the F4 frayed state and therefore the only one possessing a metastable frayed state on time scales exceeding the τ = 1.2 ns lag time of our MSMs.

### 3.6 Disruption of Repetitive AT Tracts Promotes Two-State “All-or-Nothing” Kinetics (GC-Mix)

The GC-mix sequence is the only one of the four sequences studied that exhibits simple two-state “all-or-nothing” behavior.^17,19,25,26^ This characterization is intended to reflect the mechanistic observation that GC-mix MSM comprises just two states, the hybridized H and dehybridized D (Figure 2c), indicating that association and dissociation of the strands proceeds directly without passing through any metastable intermediate states resolvable under the τ = 1.2 ns lag time of our MSM. Indeed, the sequence’s propensity to fray in simulation and the role of termini fraying in dissociation experiments^19^ indicate that there remain deviations from “all-or-nothing” behavior which are not fully captured within the MSM lag time. (We note that “all-or-nothing” can also be used, somewhat ambiguously, to refer to a two-state thermodynamic model where the hybridized and dehybridized are separated by a large free energy barrier but that the transitions between them may proceed through a network of metastable states.) The two-state behavior appears to arise as a consequence of the placement of the G:C pair that maximally disrupts the repetitive AT tract within the decamer and destabilizing either out-of-register shifted states or frayed states. We note that we do observe substantial transient fraying of the terminal two-base AT tails within our dynamical simulations, but these frayed states are not sufficiently thermodynamically stable to produce a metastable macrostate within the resulting MSM. This stands in contrast to the metastable F4 state populated by GC-core. Our MSM predicts a MFPT for D → H of 2.9 μs and for H → D of 2.3 μs, which are again nearly equal.

**Figure 4:**
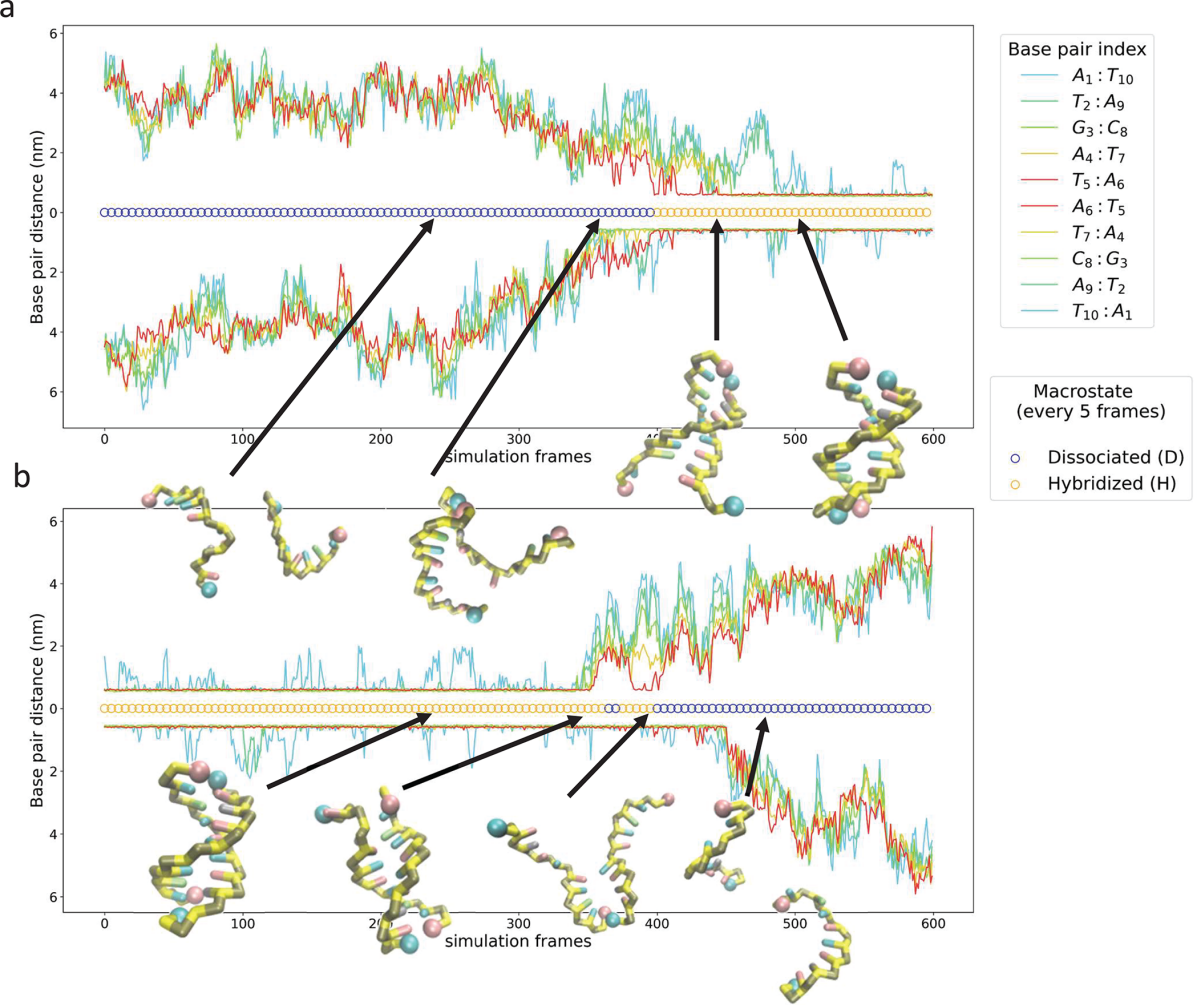
GC-mix hybridizes by nucleation–zippering and dehybridizes by fraying–peeling. Tracking of the 10 intermolecular distances between native WC base pairs over the course of an (a) hybridization event and (b) dehybridization event. Symmetrically permutable distances (e.g., A_1_:T_10_ and T_10_:A_1_) are reflected across the *x*-axis to avoid congestion in the plot. Circles superposed on the *x*-axis indicate the instantaneous MSM state assignment as dissociated D (blue) or hybridized H (orange). Hybridization tends to occur by a nucleation–zippering mechanism, wherein a native G:C pair and adjacent A:T pair or 2–3 central A:T pairs first form prior to rapid formation of the duplex. Dehybridization tends to occur by a fraying–peeling mechanism wherein fraying of the two-base AT-tails on one or both sides of the duplex precedes dissociation of the central native base pairs and complete dissolution of the duplex. Four additional hybridization events and four additional dehybridization events are presented in Figure S7.

Given the very simple two-state “all-or-nothing” behavior of GC-mix and the absence of any intermediate metastable states, we sought to interrogate our simulation trajectory data to elucidate the hybridization and dehybridization mechanisms. To do so, we followed all 10 intermolecular distances between native WC base pairs and tracked their evolution through a number of hybridization and dehybridization events. We present one representative example of each event in Figure 4 and four more in Figure S7. During the hybridization process, we observe a global decrease in all 10 distances as the strands approach one another and the formation of key native WC contacts immediately prior to duplex formation: specifically, one of the G:C WC pairs and at least one neighboring A:T pair or 2–3 central A:T pairs. This behavior is consistent with a “nucleation–zippering” mechanism as has been reported in previous studies.^16,20,37,52^ In dehybridization, we observe fraying of one half of the duplex with the strands remaining associated until the loss of the final A:T pairs and ultimately the last G:C pair. Qualitatively, we observed some short-lived states composed of two to four native WC base pair contacts immediately before full dissociation occurs, but, in contrast to the F4 state we observe in GC-core, these conformations do not constitute a metastable state within our MSM nor do they tend to reform intact duplexes. These dissociation dynamics are consistent with a “fraying–peeling” dehybridization mechanism.^32,54,55^ We observe that the principle of microscopic reversibility for a molecular system at thermodynamic equilibrium imposes the conditions of detailed balance and symmetry of the classical equations of motion under time reversibility.^129^ A consequence of these considerations is that if our system is indeed at equilibrium, then the “fraying–peeling” dehybridization mechanism can be considered a reversal of the “nucleation–zippering” hybridization pathway since the time-reversed simulation trajectories represent an equally valid sampling of the system dynamics. This is indeed borne out by our mechanistic observations for GC-mix wherein the early stages of “nucleation–zippering” proceed by the formation of one G:C contact followed by one or more additional A:T pairs and the late stages by the formation of all remaining WC pairs, which we compare with the early stages “fraying–peeling” wherein one half of the duplex frays and the late stages wherein dissociation finally completes by the dissolution of the last few A:T contacts and the final G:C pair.

### 3.7 Long-Lived Metastable Shifted States Predicted by the MSM Are Resolved by T-Jump IR

Finally, we sought to validate the predictions of our sequence-dependent MSMs against experimental T-jump IR spectroscopy. T-jump IR measurements commence from a low temperature, apply a step jump in temperature, and track the relaxation of the system to the dehybridized state. We hypothesized that the influence of the out-of-register shifted states present in the AT-all and GC-end sequences upon the system relaxation kinetics should manifest in the slow and/or fast responses measured by T-jump IR. As discussed in section 3.1, the slow IR response is largely attributed to dissociation events and the fast to terminal base fraying. With regards to the slow response, our MFPT analyses of our MSMs predict out-of-register shifting events (i.e., H → 3S2, 5S2, 3S4, 5S4) to proceed on microsecond time scales, which are commensurate with the 1.4–2.9 μs time scales for dehybridization (i.e., H → D) for each of the four sequences. As such, we anticipate that the dynamical relaxations associated with out-of-register shifted states proceed on similar time scales to, and may not be distinguishable from, the relaxation to the dehybridized state. Nevertheless, the presence of these out-of-register shifted states in the low-temperature equilibrium ensemble prior to the T-jump step may be observable via their influence on the fast T-jump IR response attributable to fraying. Specifically, we hypothesize that the dangling ends and inert tails present in the out-of-register shifted states should promote a broader fraying response over the course of the relaxation that is distinct from that of in-register fraying. This heterogeneity of configurations should lead to heterogeneous dynamics, manifested in the observation of a more stretched relaxation over experimental time scales of 70–100 ns. Analysis of the MSM equilibrium distributions (Figure 2b) reveals 10.0% of the equilibrium ensemble to reside in out-of-register shifted states 3S2, 5S2, 3S4, and 5S4 for AT-all, compared to just 0.23% for GC-end, and 0% for GC-core and GC-mix. It is our conjecture that a substantial population of out-of-register shifted states in the pre-T-jump AT-all ensemble should be distinguishable from the GC-end, GC-core, and GC-mix as an elongation of the fast relaxation response associated with terminal base fraying.

**Figure 5:**
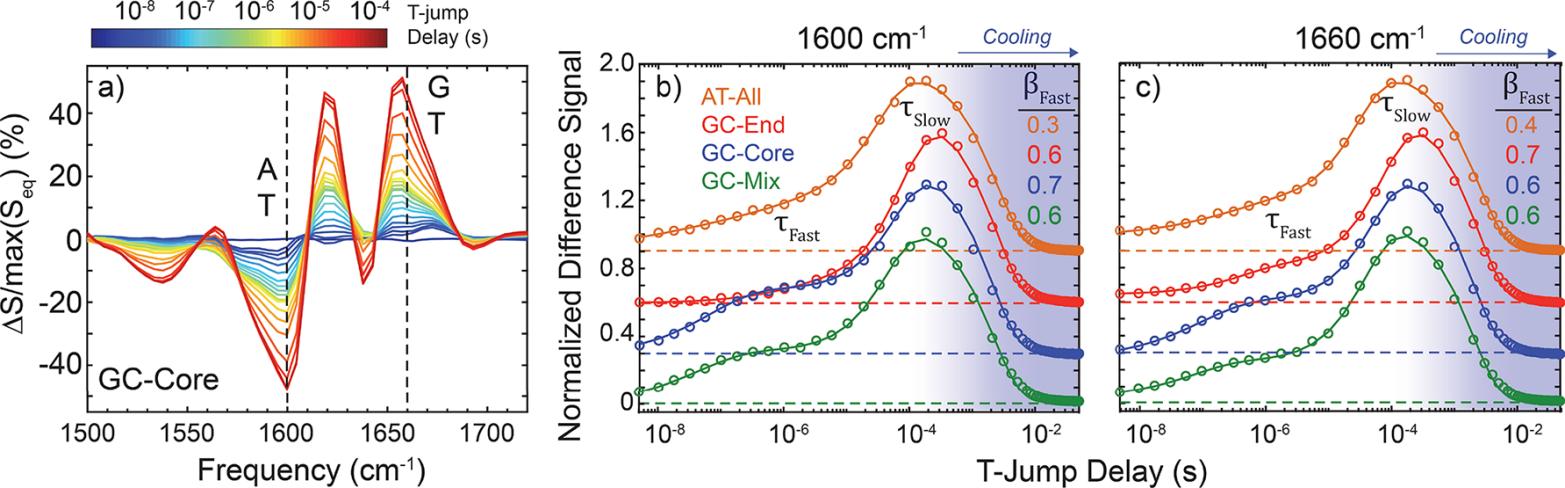
T-Jump IR responses reflect sequence-dependent conformational heterogeneity. The final temperature for each measurement is AT-all, 320 K; GC-end, 319 K; GC-core, 327 K; and GC-mix, 326 K. (a) Mid-IR t-HDVE difference spectra for GC-core at time delays from 5 ns to 560 μs. Normalized time traces for each sequence are shown at (b) 1600 cm^–1^ and (c) 1660 cm^–1^. The signal at 1600 cm^–1^ corresponds to changes in A and T ring vibrations while the signal at 1660 cm^–1^ contains contributions from G and T carbonyl vibrations. Each time trace is fit to the sum of a stretched exponential with two exponentials (solid lines): *S*(*t*) = *A* exp(−(*t*/τ_fast_)^β_fast_^) + *B* exp(−*t*/τ_slow_) + *C* exp(−*t*/τ_cool_). The stretched exponential describes the process from 5 ns to 1 μs, and the two exponentials describe the signal increase from 1 to 320 μs and signal decay from rehybridization induced by thermal relaxation back to the initial temperature. The stretch factor β_fast_ for the fits at 1600 and 1660 cm^–1^ are reported directly on the plots in panels b and c.

We present in Figure 5 our T-jump IR t-HDVE difference spectra and corresponding normalized time traces at 1600 and 1660 cm^–1^. The signal at 1600 cm^–1^ corresponds to changes in A and T ring vibrations while the signal at 1660 cm^–1^ contains contributions from G and T carbonyl vibrations. Each time trace was fitted to the sum of a stretched exponential and two exponentials *S*(*t*) = *A* exp(−(*t*/τ_fast_)^β_fast_^) + *B* exp(−*t*/τ_slow_) + *C* exp(−*t*/τ_cool_). The stretched exponential describes the relaxation process from 5 ns to 1 μs, and the two exponentials describe the signal increase from 1 to 320 μs and signal decay from rehybridization induced by thermal relaxation back to the initial temperature. The fitting parameters *A*, *B*, and *C* correspond to the relative amplitudes of the three kinetic responses and the stretch factor β_fast_ to the heterogeneity of fraying dynamics at the fast time scale. A testable prediction of our hypothesis is that the long-lived out-of-register shifted states in AT-all should result in a significantly smaller value for the fitted β_fast_ parameter (i.e., a more stretched response) relative to those for GC-end, GC-core, and GC-mix. This hypothesis was supported by the experimental time series at both 1600 cm^–1^, where β_fast_^AT-all^ = 0.3 compared to β_fast_^GC-end^ = 0.6, β_fast_^GC-core^ = 0.7, and β_fast_^GC-mix^ = 0.6, and 1660 cm^–1^, where β_fast_^AT-all^ = 0.4 compared to β_fast_^GC-end^ = 0.7, β_fast_^GC-core^ = 0.6, and β_fast_^GC-mix^ = 0.6. This result supports our hypothesis and validates a testable prediction of our sequence-dependent MSMs.

## 4 Conclusions

We have conducted an integrated computational and experimental study of the sequence-dependent kinetic mechanisms for the hybridization and dehybridization dynamics of a family of four self-complementary 10-mer DNA oligomers: ATATATATAT (AT-all), GATATATATC (GC-end), ATATGCATAT (GC-core), and ATGATATCAT (GC-mix). We conducted 1 ms of unbiased coarse-grained molecular dynamics simulations at the melting temperature of each sequence and employed deep learning techniques to construct high-resolution Markov state models as predictive and interpretable models of the sequence dependent dynamics. T-jump IR spectroscopy was used to calibrate the kinetic time scales of the coarse-grained molecular model and validate the kinetic prediction of the Markov state models that the AT-all sequence should possess long-lived out-of-register shifted states that are detectable within T-jump IR t-HDVE time traces. Our results reveal that the specific placement of interrupting G:C pairs within an otherwise repetitive AT sequence can have a profound impact on the kinetic pathways and mechanisms for association and dissociation of the DNA duplex. In particular, we found AT-all to possess the richest and most complex kinetic landscape of the four sequences that is dominated by out-of-register shifted states that participate in 33% of complete hybridization events—pathways leading from the dissociated state to full duplex formation—and dehybridization events—pathways from the complete duplex to full dissociation. Introduction of the G:C pairs at the end of the strand maintains an eight-base-pair repetitive AT tract and the GC-end kinetic landscape possess all but one of the same out-of-register shifted states as AT-all. Destabilization of the GC-end shifted states relative to AT-all, however, results in a far more limited participation of these states with only 7% of GC-end hybridization and dehybridization events passing through one or more shifted states. Placing the G:C pairs in the center of the strand maintains two four-base AT tracts on either side of the core and results in qualitatively different kinetic behaviors for GC-core. In this case, no metastable out-of-register shifted states are registered by our model with the hybridization and dehybridization pathways all passing through a strongly metastable frayed state in which one or other of the four-base AT-tracts is unbound to produce two free AT-tails. Finally, placing the G:C bases between the center and end of the strand to maximally disrupt the repetitive AT tracts results in no metastable out-of-register or frayed states for GC-mix and results in simple two-state “all-or-nothing” hybridization/dehybridization behavior. Analysis of the specific pathways reveals hybridization to largely proceed by a nucleation-zippering mechanism and dehybridization to proceed by a fraying-peeling mechanism.

The ordering of the computationally predicted kinetic landscapes from most to least complex—AT-all > GC-end > GC-core > GC-mix—is largely dictated by sequence repetitiveness, specifically the number of consecutive AT motifs. We note that this ordering differs from our previously reported ordering in terms of deviation from two-state behavior of GC-core > GC-mix > AT-all > GC-end.^19,28^ We can understand these two apparently discrepant orderings by understanding that the latter was deduced based on experimental analyses and lattice models that did not account for out-of-register states and focused largely on fraying behaviors. Indeed, under the assumption that fraying is the dominant kinetic process relative to out-of-register shifting, we can harmonize the predictions of the present work with our prior work by eliminating all out-of-register shifted states in our fitted MSMs (Figure 2c), in which case we find GC-core to contain an F4 frayed intermediate and the remaining sequences to all have simple two-state dynamics such that the predicted ordering is GC-core > GC-mix ≈ AT-all ≈ GC-end.

In sum, our results demonstrate the profound effect of sequence upon the kinetic landscapes, metastable states, and hybridization/dehybridization mechanisms of short DNA oligomers. Our analysis of this small family of sequences expose preliminary design principles for the (meta)stability of out-of-register and frayed states but we anticipate much greater richness in the landscapes will emerge with studies of longer and more diverse sequences. Going forward, we will extend this work to discern more general trends in sequence-dependent hybridization/dehybridization for a wider range of oligomer sequences and motivate strategies for experimental comparisons. We also suggest that the MSM approach followed in this work, possibly coupled with biased sampling and reweighting techniques,^94,130^ may be well-suited to expose changes in the hybridization/dehybridization mechanism and kinetics as a function of temperature. We anticipate that these insights may provide foundational design rules by which to improve understanding of *in vivo* hybridization processes and rationally engineer optimized sequences for DNA nanotechnology applications such as DNA-PAINT^7^ and DNA barcoding.^6^

## Supporting Information Available

Details of nearest neighbor (NN) model calculations, supplementary figures illustrating selection of optimal number of SRV slow modes for each sequence, convergence of MSM implied time scales, MSM Chapman–Kolmogorov tests, “computational T-jump” calculations, and GC-mix hybridization and dehybridization trajectories(PDF)

## Notes

Input files and analysis scripts have been uploaded to Github (https://github.com/mrjoness/Oligo-MSMs) and 45 GB of featurized trajectories and experimental data to Zenodo (10.5281/zenodo.5148949).

## References

[ref1] Seeman N. C.; Sleiman H. F. DNA Nanotechnology. Nat. Rev. Mater. 2018, 3, 17068 10.1038/natrevmats.2017.68.

[ref2] Adleman L. Molecular Computation of Solutions to Combinatorial Problems. Science 1994, 266, 1021–1024. 10.1126/science.7973651. 7973651

[ref3] Rothemund P. W. Folding DNA to Create Nanoscale Shapes and Patterns. Nature 2006, 440, 297–302. 10.1038/nature04586. 16541064

[ref4] Gu H.; Chao J.; Xiao S. J.; Seeman N. C. A Proximity-based Programmable DNA Nanoscale Assembly Line. Nature 2010, 465, 202–205. 10.1038/nature09026. 20463734 PMC2872101

[ref5] Schnitzbauer J.; Strauss M. T.; Schlichthaerle T.; Schueder F.; Jungmann R. Super-resolution Microscopy with DNA-PAINT. Nat. Protoc. 2017, 12, 1198–1228. 10.1038/nprot.2017.024. 28518172

[ref6] Shah S.; Dubey A. K.; Reif J. Improved Optical Multiplexing with Temporal DNA Barcodes. ACS Synth. Biol. 2019, 8, 1100–1111. 10.1021/acssynbio.9b00010. 30951289

[ref7] Strauss S.; Jungmann R. Up to 100-fold Speed-up and Multiplexing in Optimized DNA-PAINT. Nat. Methods 2020, 17, 789–791. 10.1038/s41592-020-0869-x. 32601424 PMC7610413

[ref8] SantaLucia J. A Unified View of Polymer, Dumbbell, and Oligonucleotide DNA Nearest-Neighbor Thermodynamics. Proc. Natl. Acad. Sci. U. S. A. 1998, 95, 1460–1465. 10.1073/pnas.95.4.1460. 9465037 PMC19045

[ref9] Santalucia J.; Hicks D. The Thermodynamics of DNA Structural Motifs. Annu. Rev. Biophys. Biomol. Struct. 2004, 33, 415–440. 10.1146/annurev.biophys.32.110601.141800. 15139820

[ref10] Tsukanov R.; Tomov T. E.; Masoud R.; Drory H.; Plavner N.; Liber M.; Nir E. Detailed Study of DNA Hairpin Dynamics using Single-Molecule Fluorescence Assisted by DNA Origami. J. Phys. Chem. B 2013, 117, 11932–11942. 10.1021/jp4059214. 24041226

[ref11] Mosayebi M.; Romano F.; Ouldridge T. E.; Louis A. A.; Doye J. P. The Role of Loop Stacking in the Dynamics of DNA Hairpin Formation. J. Phys. Chem. B 2014, 118, 14326–14335. 10.1021/jp510061f. 25404188

[ref12] Mergny J. L.; Sen D. DNA Quadruple Helices in Nanotechnology. Chem. Rev. 2019, 119, 6290–6325. 10.1021/acs.chemrev.8b00629. 30605316

[ref13] Deluca M.; Shi Z.; Castro C. E.; Arya G. Dynamic DNA Nanotechnology: Toward Functional Nanoscale Devices. Nanoscale Horiz. 2020, 5, 182–201. 10.1039/C9NH00529C.

[ref14] Cordes T.; Santoso Y.; Tomescu A. I.; Gryte K.; Hwang L. C.; Camará B.; Wigneshweraraj S.; Kapanidis A. N. Sensing DNA Opening in Transcription Using Quenchable Förster Resonance Energy Transfer. Biochemistry 2010, 49, 9171–9180. 10.1021/bi101184g. 20818825

[ref15] Naimark O.B.; Bayandin Y. V.; Beloglazova Y. A.; Gagarskich O.N.; Grishko V.V.; Nikitiuk A.S.; Voronina A.O. DNA Transformation, Cell Epigenetic Landscape and Open Complex Dynamics in Cancer Development. Math. Biol. Bioinf. 2020, 15, 251–267. 10.17537/2020.15.251.

[ref16] Yin Y.; Zhao X. S. Kinetics and Dynamics of DNA Hybridization. Acc. Chem. Res. 2011, 44, 1172–1181. 10.1021/ar200068j. 21718008

[ref17] Xiao S.; Sharpe D. J.; Chakraborty D.; Wales D. J. Energy Landscapes and Hybridization Pathways for DNA Hexamer Duplexes. J. Phys. Chem. Lett. 2019, 10, 6771–6779. 10.1021/acs.jpclett.9b02356. 31609632

[ref18] Hinckley D. M.; Lequieu J. P.; De Pablo J. J. Coarse-grained modeling of DNA oligomer hybridization: Length, sequence, and salt effects. J. Chem. Phys. 2014, 141, 035102 10.1063/1.4886336. 25053341

[ref19] Sanstead P. J.; Stevenson P.; Tokmakoff A. Sequence-Dependent Mechanism of DNA Oligonucleotide Dehybridization Resolved through Infrared Spectroscopy. J. Am. Chem. Soc. 2016, 138, 11792–11801. 10.1021/jacs.6b05854. 27519555

[ref20] Pörschke D.; Eigen Cooperative Nonenzymic Base Recognition III. Kinetics of the Helix-Coil Transition. J. Mol. Biol. 1971, 62, 361–381. 10.1016/0022-2836(71)90433-5. 5138337

[ref21] Zhang D. Y.; Winfree E. Control of DNA Strand Displacement Kinetics Using Toehold Exchange. J. Am. Chem. Soc. 2009, 131, 17303–17314. 10.1021/ja906987s. 19894722

[ref22] Shah S.; Dubey A. K.; Reif J. Programming Temporal DNA Barcodes for Single-Molecule Fingerprinting. Nano Lett. 2019, 19, 2668–2673. 10.1021/acs.nanolett.9b00590. 30896178

[ref23] Schickinger M.; Zacharias M.; Dietz H. Tethered Multifluorophore Motion Reveals Equilibrium Transition Kinetics of Single DNA Double Helices. Proc. Natl. Acad. Sci. U. S. A. 2018, 115, E7512–E7521. 10.1073/pnas.1800585115. 30037988 PMC6094131

[ref24] Zhang J. X.; Fang J. Z.; Duan W.; Wu L. R.; Zhang A. W.; Dalchau N.; Yordanov B.; Petersen R.; Phillips A.; Zhang D. Y. Predicting DNA Hybridization Kinetics from Sequence. Nat. Chem. 2018, 10, 91–98. 10.1038/nchem.2877. 29256499 PMC5739081

[ref25] Araque J. C.; Robert M. A. Lattice Model of Oligonucleotide Hybridization in Solution. II. Specificity and Cooperativity. J. Chem. Phys. 2016, 144, 125101 10.1063/1.4943577. 27036478

[ref26] Sikora J. R.; Rauzan B.; Stegemann R.; Deckert A. Modeling Stopped-Flow Data for Nucleic Acid Duplex Formation Reactions: The Importance of Off-Path Intermediates. J. Phys. Chem. B 2013, 117, 8966–8976. 10.1021/jp404550a. 23902467

[ref27] Wyer J. A.; Kristensen M. B.; Jones N. C.; Hoffmann S. V.; Nielsen S. B. Kinetics of DNA Duplex Formation: A-Tracts versus AT-Tracts. Phys. Chem. Chem. Phys. 2014, 16, 18827–18839. 10.1039/C4CP02252A. 25078080

[ref28] Sanstead P. J.; Tokmakoff A. Direct Observation of Activated Kinetics and Downhill Dynamics in DNA Dehybridization. J. Phys. Chem. B 2018, 122, 3088–3100. 10.1021/acs.jpcb.8b01445. 29504399

[ref29] Maciejczyk M.; Spasic A.; Liwo A.; Scheraga H. A. DNA Duplex Formation with a Coarse-Grained Model. J. Chem. Theory Comput. 2014, 10, 5020–5035. 10.1021/ct4006689. 25400520 PMC4230386

[ref30] Ouldridge T. E.; Šulc P.; Romano F.; Doye J. P. K.; Louis A. A. DNA Hybridization Kinetics: Zippering, Internal Displacement and Sequence Dependence. Nucleic Acids Res. 2013, 41, 8886–8895. 10.1093/nar/gkt687. 23935069 PMC3799446

[ref31] Flamm C.; Fontana W.; Hofacker I. L.; Schuster P. RNA folding at elementary step resolution. RNA 2000, 6, 325–338. 10.1017/S1355838200992161. 10744018 PMC1369916

[ref32] Zgarbová M.; Otyepka M.; Šponer J.; Lankaš F.; Jurečka P. Base Pair Fraying in Molecular Dynamics Simulations of DNA and RNA. J. Chem. Theory Comput. 2014, 10, 3177–3189. 10.1021/ct500120v. 26588288

[ref33] Nonin S.; Leroy J. L.; Guéron M. Terminal Base Pairs of Oligodeoxynucleotides: Imino Proton Exchange and Fraying. Biochemistry 1995, 34, 10652–10659. 10.1021/bi00033a041. 7654719

[ref34] Nikolova E. N.; Bascom G. D.; Andricioaei I.; Al-Hashimi H. M. Probing Sequence-Specific DNA Flexibility in A-Tracts and Pyrimidine-Purine Steps by Nuclear Magnetic Resonance 13C Relaxation and Molecular Dynamics Simulations. Biochemistry 2012, 51, 8654–8664. 10.1021/bi3009517. 23035755 PMC3676944

[ref35] Andreatta D.; Sen S.; Pérez Lustres J. L.; Kovalenko S. A.; Ernsting N. P.; Murphy C. J.; Coleman R. S.; Berg M. A. Ultrafast Dynamics in DNA: “Fraying” at the End of the Helix. J. Am. Chem. Soc. 2006, 128, 6885–6892. 10.1021/ja0582105. 16719468 PMC2528932

[ref36] Morrison L. E.; Stols L. M. Sensitive Fluorescence-Based Thermodynamic and Kinetic Measurements of DNA Hybridization in Solution. Biochemistry 1993, 32, 3095–3104. 10.1021/bi00063a022. 8457571

[ref37] Wetmur J. G.; Davidson N. Kinetics of Renaturation of DNA. J. Mol. Biol. 1968, 31, 349–370. 10.1016/0022-2836(68)90414-2. 5637197

[ref38] Craig M. E.; Crothers D. M.; Doty P. Relaxation Kinetics of Dimer Formation by Self Complementary Oligonucleotides. J. Mol. Biol. 1971, 62, 383–401. 10.1016/0022-2836(71)90434-7. 5138338

[ref39] Pörschke D.; Uhlenbeck O. C.; Martin F. H. Thermodynamics and Kinetics of the Helix-Coil Transition of Oligomers Containing GC Base Pairs. Biopolymers 1973, 12, 1313–1335. 10.1002/bip.1973.360120609.

[ref40] Williams A. P.; Longfellow C. E.; Freier S. M.; Kierzek R.; Turner D. H. Laser Temperature-Jump, Spectroscopic, and Thermodynamic Study of Salt Effects on Duplex Formation by dGCATGC. Biochemistry 1989, 28, 4283–4291. 10.1021/bi00436a025. 2765487

[ref41] Narayanan R.; Zhu L.; Velmurugu Y.; Roca J.; Kuznetsov S. V.; Prehna G.; Lapidus L. J.; Ansari A. Exploring the Energy Landscape of Nucleic Acid Hairpins Using Laser Temperature-Jump and Microfluidic Mixing. J. Am. Chem. Soc. 2012, 134, 18952–18963. 10.1021/ja301218e. 23078026

[ref42] Chen C.; Wang W.; Wang Z.; Wei F.; Zhao X. S. Influence of Secondary Structure on Kinetics and Reaction Mechanism of DNA Hybridization. Nucleic Acids Res. 2007, 35, 2875–2884. 10.1093/nar/gkm177. 17430963 PMC1888818

[ref43] Liu C.; Obliosca J. M.; Liu Y. L.; Chen Y. A.; Jiang N.; Yeh H. C. 3D Single-Molecule Tracking Enables Direct Hybridization Kinetics Measurement in Solution. Nanoscale 2017, 9, 5664–5670. 10.1039/C7NR01369H. 28422238 PMC5515391

[ref44] Chen X.; Zhou Y.; Qu P.; Zhao X. S. Base-by-Base Dynamics in DNA Hybridization Probed by Fluorescence Correlation Spectroscopy. J. Am. Chem. Soc. 2008, 130, 16947–16952. 10.1021/ja804628x. 19053418

[ref45] Dupuis N. F.; Holmstrom E. D.; Nesbitt D. J. Single-Molecule Kinetics Reveal Cation-Promoted DNA Duplex Formation Through Ordering of Single-Stranded Helices. Biophys. J. 2013, 105, 756–766. 10.1016/j.bpj.2013.05.061. 23931323 PMC3736743

[ref46] Sanstead P. J.; Tokmakoff A. A Lattice Model for the Interpretation of Oligonucleotide Hybridization Experiments. J. Chem. Phys. 2019, 150, 185104 10.1063/1.5092526. 31091913

[ref47] Piana S. Atomistic Simulation of the DNA Helix-Coil Transition. J. Phys. Chem. A 2007, 111, 12349–12354. 10.1021/jp0756552. 17990856

[ref48] Zerze G. H.; Stillinger F. H.; Debenedetti P. G. Thermodynamics of DNA Hybridization from Atomistic Simulations. J. Phys. Chem. B 2021, 125, 771–779. 10.1021/acs.jpcb.0c09237. 33434025

[ref49] Hinckley D. M.; Freeman G. S.; Whitmer J. K.; De Pablo J. J. An Experimentally-Informed Coarse-Grained 3-Site-Per-Nucleotide Model of DNA: Structure, Thermodynamics, and Dynamics of Hybridization. J. Chem. Phys. 2013, 139, 144903 10.1063/1.4822042. 24116642 PMC3808442

[ref50] Schmitt T. J.; Rogers J. B.; Knotts T. A. IV Exploring the Mechanisms of DNA Hybridization on a Surface. J. Chem. Phys. 2013, 138, 035102 10.1063/1.4775480. 23343305

[ref51] Sambriski E. J.; Schwartz D. C.; De Pablo J. J. Uncovering Pathways in DNA Oligonucleotide Hybridization via Transition State Analysis. Proc. Natl. Acad. Sci. U. S. A. 2009, 106, 18125–18130. 10.1073/pnas.0904721106. 19815517 PMC2759370

[ref52] Sambriski E. J.; Ortiz V.; De Pablo J. J. Sequence Effects in the Melting and Renaturation of Short DNA Oligonucleotides: Structure and Mechanistic Pathways. J. Phys.: Condens. Matter 2009, 21, 034105 10.1088/0953-8984/21/3/034105. 21817250 PMC3886633

[ref53] Hoefert M. J.; Sambriski E. J.; José De Pablo J. Molecular Pathways in DNA-DNA Hybridization of Surface-Bound Oligonucleotides. Soft Matter 2011, 7, 560–566. 10.1039/C0SM00729C.

[ref54] Wong K. Y.; Pettitt B. M. The Pathway of Oligomeric DNA Melting Investigated by Molecular Dynamics Simulations. Biophys. J. 2008, 95, 5618–5626. 10.1529/biophysj.108.141010. 18952784 PMC2599842

[ref55] Perez A.; Orozco M. Real-Time Atomistic Description of DNA Unfolding. Angew. Chem., Int. Ed. 2010, 49, 4805–4808. 10.1002/anie.201000593. 20480472

[ref56] Gallicchio E.; Andrec M.; Felts A. K.; Levy R. M. Temperature weighted histogram analysis method, replica exchange, and transition paths. J. Phys. Chem. B 2005, 109, 6722–6731. 10.1021/jp045294f. 16851756

[ref57] Souaille M.; Roux B. Extension to the weighted histogram analysis method: Combining umbrella sampling with free energy calculations. Comput. Phys. Commun. 2001, 135, 40–57. 10.1016/S0010-4655(00)00215-0.

[ref58] Shirts M. R.; Chodera J. D. Statistically optimal analysis of samples from multiple equilibrium states. J. Chem. Phys. 2008, 129, 124105 10.1063/1.2978177. 19045004 PMC2671659

[ref59] Kumar S.; Rosenberg J. M.; Bouzida D.; Swendsen R. H.; Kollman P. A. THE weighted histogram analysis method for free–energy calculations on biomolecules. I. The method. J. Comput. Chem. 1992, 13, 1011–1021. 10.1002/jcc.540130812.

[ref60] Prinz J. H.; Chodera J. D.; Pande V. S.; Swope W. C.; Smith J. C.; Noé F. Optimal Use of Data in Parallel Tempering Simulations for the Construction of Discrete-State Markov models of Biomolecular Dynamics. J. Chem. Phys. 2011, 134, 244108 10.1063/1.3592153. 21721613 PMC3139503

[ref61] Chodera J. D.; Swope W. C.; Noé F.; Prinz J. H.; Shirts M. R.; Pande V. S. Dynamical reweighting: Improved Estimates of Dynamical Properties from Simulations at Multiple Temperatures. J. Chem. Phys. 2011, 134, 244107 10.1063/1.3592152. 21721612 PMC3143679

[ref62] Stelzl L. S.; Kells A.; Rosta E.; Hummer G. Dynamic Histogram Analysis To Determine Free Energies and Rates from Biased Simulations. J. Chem. Theory Comput. 2017, 13, 6328–6342. 10.1021/acs.jctc.7b00373. 29059525

[ref63] Donati L.; Hartmann C.; Keller B. G. Girsanov Reweighting for Path Ensembles and Markov State Models. arXiv Preprint (Condensed Matter, Statistical Mechanics) https://arxiv.org/abs/1703.05498 (accessed 2021-09-27).

[ref64] Donati L.; Keller B. G. Girsanov Reweighting for Metadynamics Simulations. J. Chem. Phys. 2018, 149, 072335 10.1063/1.5027728. 30134671

[ref65] Quer J.; Donati L.; Keller B. G.; Weber M. An Automatic Adaptive Importance Sampling Algorithm for Molecular Dynamics in Reaction Coordinates. SIAM J. SCI. Comput 2018, 40, A653–A670. 10.1137/17M1124772.

[ref66] Mey A. S.; Wu H.; Noé F. xTRAM: Estimating equilibrium expectations from time-correlated simulation data at multiple thermodynamic states. Phys. Rev. X 2014, 4, 041018 10.1103/PhysRevX.4.041018.

[ref67] Markegard C. B.; Fu I. W.; Reddy K. A.; Nguyen H. D. Coarse-Grained Simulation Study of Sequence Effects on DNA Hybridization in a Concentrated Environment. J. Phys. Chem. B 2015, 119, 1823–1834. 10.1021/jp509857k. 25581253

[ref68] Dans P. D.; Walther J.; Gómez H.; Orozco M. Multiscale Simulation of DNA. Curr. Opin. Struct. Biol. 2016, 37, 29–45. 10.1016/j.sbi.2015.11.011. 26708341

[ref69] Lequieu J.; Córdoba A.; Schwartz D. C.; De Pablo J. J. Tension-Dependent Free Energies of Nucleosome Unwrapping. ACS Cent. Sci. 2016, 2, 660–666. 10.1021/acscentsci.6b00201. 27725965 PMC5043429

[ref70] Lequieu J.; Schwartz D. C.; De Pablo J. J. In Silico Evidence for Sequence-Dependent Nucleosome Sliding. Proc. Natl. Acad. Sci. U. S. A. 2017, 114, E9197–E9205. 10.1073/pnas.1705685114. 29078285 PMC5676884

[ref71] Terakawa T.; Takada S. P53 Dynamics Upon Response Element Recognition Explored by Molecular Simulations. Sci. Rep. 2015, 5, 17107 10.1038/srep17107. 26596470 PMC4656996

[ref72] Tan C.; Takada S. Dynamic and Structural Modeling of the Specificity in Protein-DNA Interactions Guided by Binding Assay and Structure Data. J. Chem. Theory Comput. 2018, 14, 3877–3889. 10.1021/acs.jctc.8b00299. 29806939

[ref73] Srinivas N.; Ouldridge T. E.; Šulc P.; Schaeffer J. M.; Yurke B.; Louis A. A.; Doye J. P.; Winfree E. On the Biophysics and Kinetics of Toehold-Mediated DNA Strand Displacement. Nucleic Acids Res. 2013, 41, 10641–10658. 10.1093/nar/gkt801. 24019238 PMC3905871

[ref74] Haley N. E.; Ouldridge T. E.; Mullor Ruiz I.; Geraldini A.; Louis A. A.; Bath J.; Turberfield A. J. Design of hidden Thermodynamic Driving for Non-Equilibrium Systems via Mismatch Elimination During DNA Strand Displacement. Nat. Commun. 2020, 11, 2562 10.1038/s41467-020-16353-y. 32444600 PMC7244503

[ref75] Snodin B. E.; Schreck J. S.; Romano F.; Louis A. A.; Doye J. P. Coarse-Grained Modelling of the Structural Properties of DNA Origami. Nucleic Acids Res. 2019, 47, 1585–1597. 10.1093/nar/gky1304. 30605514 PMC6379721

[ref76] Doye J. P.; Fowler H.; Prešern D.; Bohlin J.; Rovigatti L.; Romano F.; Šulc P.; Wong C. K.; Louis A. A.; Schreck J. S. The oxDNA Coarse-Grained Model as a Tool to Simulate DNA Origami. Submitted: 10 Apr 2020. arXiv Preprint (Soft Condensed Matter) https://arxiv.org/abs/2004.05052 (accessed 2021-05-20). 10.1007/978-1-0716-3028-0_6 37166713

[ref77] Córdoba A.; Hinckley D. M.; Lequieu J.; de Pablo J. J. A Molecular View of the Dynamics of dsDNA Packing Inside Viral Capsids in the Presence of Ions. Biophys. J. 2017, 112, 1302–1315. 10.1016/j.bpj.2017.02.015. 28402874 PMC5389966

[ref78] Lu W.; Bueno C.; Schafer N. P.; Moller J.; Jin S.; Chen X.; Chen M.; Gu X.; Davtyan A.; de Pablo J. J.; Wolynes P. G. OpenAWSEM with Open3SPN2: A Fast, Flexible, and Accessible Framework for Large-Scale Coarse-Grained Biomolecular Simulations. PLoS Comput. Biol. 2021, 17, e1008308 10.1371/journal.pcbi.1008308. 33577557 PMC7906472

[ref79] Plimpton S. Fast Parallel Algorithms for Short–Range Molecular Dynamics. J. Comput. Phys. 1995, 117, 1–19. 10.1006/jcph.1995.1039.

[ref80] Dunweg W.; Paul B. Brownian Dynamics Simulations Without Gaussian Random Numbers. Int. J. Mod. Phys. C 1991, 2, 817–827. 10.1142/S0129183191001037.

[ref81] Bussi G.; Parrinello M. Accurate Sampling Using Langevin Dynamics. Phys. Rev. E 2007, 75, 056707 10.1103/PhysRevE.75.056707. 17677198

[ref82] Nkodo A. E.; Garnier J. M.; Tinland B.; Ren H.; Desruisseaux C.; McCormick L. C.; Drouin G.; Slater G. W. Diffusion Coefficient of DNA Molecules During Free Solution Electrophoresis. Electrophoresis 2001, 22, 2424–2432. 10.1002/1522-2683(200107)22:12<2424::AID-ELPS2424>3.0.CO;2-1. 11519946

[ref83] Debye P.; Huckel E. Zur theorie der elektrolyte. II. Das Grenzgesetz für die elektrische Leitfähigkeit. Phys. 1923, 24, 305–328.

[ref84] Schneider T.; Stoll E. Molecular-dynamics Study of a Three-Dimensional One-Component Model for Distortive Phase Transitions. Phys. Rev. B: Condens. Matter Mater. Phys. 1978, 17, 1302–1322. 10.1103/PhysRevB.17.1302.

[ref85] Arnott S.; Smith P. J. C.; Chandrasekaran Handbook of Biochemistry and Molecular Biology; CRC Press, 1976; pp 411–422.

[ref86] Sengupta U.; Carballo-pacheco M.; Strodel B. Automated Markov State Models for Molecular Dynamics Simulations of Aggregation and Self-Assembly. J. Chem. Phys. 2019, 150, 115101 10.1063/1.5083915. 30901988

[ref87] Pande V. S.; Beauchamp K.; Bowman G. R. Everything You Wanted to Know About Markov State Models But Were Afraid to Ask. Methods 2010, 52, 99–105. 10.1016/j.ymeth.2010.06.002. 20570730 PMC2933958

[ref88] Chodera J. D.; Noé F. Markov State Models of Biomolecular Conformational Dynamics. Curr. Opin. Struct. Biol. 2014, 25, 135–144. 10.1016/j.sbi.2014.04.002. 24836551 PMC4124001

[ref89] Husic B. E.; Pande V. S. Markov State Models: From an Art to a Science. J. Am. Chem. Soc. 2018, 140, 2386–2396. 10.1021/jacs.7b12191. 29323881

[ref90] Wehmeyer C.; Scherer M. K.; Hempel T.; Husic B. E.; Olsson S.; Noé F. Introduction to Markov State Modeling with the PyEMMA Software. Living J. Comput. Mol. Sci. 2019, 1, 5965 10.33011/livecoms.1.1.5965.

[ref91] Jin R.; Maibaum L. Mechanisms of DNA Hybridization: Transition Path Analysis of a Simulation-Informed Markov Model. J. Chem. Phys. 2019, 150, 105103 10.1063/1.5054593. 30876357

[ref92] Pinamonti G.; Zhao J.; Condon D. E.; Paul F.; Noe F.; Turner D. H.; Bussi G. Predicting the Kinetics of RNA Oligonucleotides Using Markov State Models. J. Chem. Theory Comput. 2017, 13, 926–934. 10.1021/acs.jctc.6b00982. 28001394 PMC5450499

[ref93] Sidky H.; Chen W.; Ferguson A. L. High-Resolution Markov State Models for the Dynamics of Trp-Cage Miniprotein Constructed over Slow Folding Modes Identified by State-Free Reversible VAMPnets. J. Phys. Chem. B 2019, 123, 7999–8009. 10.1021/acs.jpcb.9b05578. 31453697

[ref94] Scherer M. K.; Trendelkamp-Schroer B.; Paul F.; Perez-Hernandez G.; Hoffmann M.; Plattner N.; Wehmeyer C.; Prinz J.-H.; Noe F. PyEMMA 2: A Software Package for Estimation, Validation, and Analysis of Markov Models. J. Chem. Theory Comput. 2015, 11, 5525–5542. 10.1021/acs.jctc.5b00743. 26574340

[ref95] McGibbon R. T.; Beauchamp K. A.; Harrigan M. P.; Klein C.; Swails J. M.; Hernández C. X.; Schwantes C. R.; Wang L. P.; Lane T. J.; Pande V. S. MDTraj: A Modern Open Library for the Analysis of Molecular Dynamics Trajectories. Biophys. J. 2015, 109, 1528–1532. 10.1016/j.bpj.2015.08.015. 26488642 PMC4623899

[ref96] Noé F.; Nüske F. A Variational Approach to Modeling Slow Processes in Stochastic Dynamical Systems. Multiscale Model. Simul. 2013, 11, 635–655. 10.1137/110858616.

[ref97] Noé F.; Clementi C. Kinetic Distance and Kinetic Maps from Molecular Dynamics Simulation. J. Chem. Theory Comput. 2015, 11, 5002–5011. 10.1021/acs.jctc.5b00553. 26574285

[ref98] Scherer M. K.; Husic B. E.; Hoffmann M.; Paul F.; Wu H.; Noé F. Variational Selection of Features for Molecular Kinetics. J. Chem. Phys. 2019, 150, 194108 10.1063/1.5083040. 31117766

[ref99] Wu H.; Noé F. Variational Approach for Learning Markov Processes from Time Series Data. J. Nonlinear Sci. 2020, 30, 23–66. 10.1007/s00332-019-09567-y.

[ref100] Pérez-Hernández G.; Paul F.; Giorgino T.; De Fabritiis G.; Noé F. Identification of Slow Molecular Order Parameters for Markov Model Construction. J. Chem. Phys. 2013, 139, 015102 10.1063/1.4811489. 23822324

[ref101] Schwantes C. R.; Pande V. S. Improvements in Markov State Model Construction Reveal Many Non-Native Interactions in the Folding of NTL9. J. Chem. Theory Comput. 2013, 9, 2000–2009. 10.1021/ct300878a. 23750122 PMC3673732

[ref102] Chen W.; Sidky H.; Ferguson A. L. Nonlinear Discovery of Slow Molecular Modes using State-Free Reversible VAMPnets. J. Chem. Phys. 2019, 150, 214114 10.1063/1.5092521. 31176319

[ref103] Prinz J.-H.; Wu H.; Sarich M.; Keller B.; Senne M.; Held M.; Chodera J. D.; Schütte C.; Noé F. Markov Models of Molecular Kinetics: Generation and Validation. J. Chem. Phys. 2011, 134, 174105 10.1063/1.3565032. 21548671

[ref104] Husic B. E.; Pande V. S. Note: MSMLag Time Cannot Be Used for Variational Model Selection. J. Chem. Phys. 2017, 147, 176101 10.1063/1.5002086. 29117698 PMC5669980

[ref105] Röblitz S.; Weber M. Fuzzy Spectral Clustering by PCCA+: Application to Markov State Models and Data Classification. Adv. Data Anal. Classif. 2013, 7, 147–179. 10.1007/s11634-013-0134-6.

[ref106] Weber M. Implications of PCCA+ in Molecular Simulation. Computation 2018, 6, 20 10.3390/computation6010020.

[ref107] Kube S.; Weber M. A Coarse Graining Method for the Identification of Transition Rates Between Bolecular Conformations. J. Chem. Phys. 2007, 126, 024103 10.1063/1.2404953. 17228939

[ref108] Noé F.; Schütte C.; Vanden-Eijnden E.; Reich L.; Weikl T. R. Constructing the Equilibrium Ensemble of Folding Pathways From Short Off-Equilibrium Simulations. Proc. Natl. Acad. Sci. U. S. A. 2009, 106, 19011–19016. 10.1073/pnas.0905466106. 19887634 PMC2772816

[ref109] Chung H. S.; Khalil M.; Smith A. W.; Tokmakoff A. Transient Two-Dimensional IR Spectrometer for Probing Nanosecond Temperature-Jump Kinetics. Rev. Sci. Instrum. 2007, 78, 063101 10.1063/1.2743168. 17614599

[ref110] Jones K. C.; Ganim Z.; Tokmakoff A. Heterodyne-Detected Dispersed Vibrational Echo Spectroscopy. J. Phys. Chem. A 2009, 113, 14060–14066. 10.1021/jp906256s. 19938867

[ref111] Jones K. C.; Ganim Z.; Peng C. S.; Tokmakoff A. Transient Two-Dimensional Spectroscopy with Linear Absorption Corrections Applied to Temperature-Jump Two-Dimensional Infrared. J. Opt. Soc. Am. B 2012, 29, 118–129. 10.1364/JOSAB.29.000118.

[ref112] Kumar A. T.; Zhu L.; Christian J. F.; Demidov A. A.; Champion P. M. On the Rate Distribution Analysis of Kinetic Data Using the Maximum Entropy Method: Applications to Myoglobin Relaxation on the Nanosecond and Femtosecond Timescales. J. Phys. Chem. B 2001, 105, 7847–7856. 10.1021/jp0101209.

[ref113] Bernasconi C. Relaxation Kinetics; Elsevier, 2012.

[ref114] Marky L. A.; Breslauer K. J. Calculating Thermodynamic Data for Transitions of any Molecularity from Equilibrium Melting Curves. Biopolymers 1987, 26, 1601–1620. 10.1002/bip.360260911. 3663875

[ref115] Galindo-Murillo R.; Roe D. R.; Cheatham T. E. Convergence and Reproducibility in Molecular Dynamics Simulations of the DNA Duplex d(GCACGAACGAACGAACGC). Biochim. Biophys. Acta, Gen. Subj. 2015, 1850, 1041–1058. 10.1016/j.bbagen.2014.09.007. PMC4339415 25219455

[ref116] Pinamonti G.; Paul F.; Noe F.; Rodriguez A.; Bussi G. The Mechanism of RNA Base Fraying: Molecular Dynamics Simulations Analyzed with Core-Set Markov State Models. J. Chem. Phys. 2019, 150, 154123 10.1063/1.5083227. 31005065

[ref117] Marrink S. J.; Risselada H. J.; Yefimov S.; Tieleman D. P.; De Vries A. H. The MARTINI Force Field: Coarse Grained Model for Biomolecular Simulations. J. Phys. Chem. B 2007, 111, 7812–7824. 10.1021/jp071097f. 17569554

[ref118] Fritz D.; Koschke K.; Harmandaris V. A.; Van Der Vegt N. F.; Kremer K. Multiscale Modeling of Soft Matter: Scaling of Dynamics. Phys. Chem. Chem. Phys. 2011, 13, 10412–10420. 10.1039/c1cp20247b. 21468407

[ref119] Marrink S. J.; Tieleman D. P. Perspective on the Martini Model. Chem. Soc. Rev. 2013, 42, 6801–6822. 10.1039/c3cs60093a. 23708257

[ref120] Owczarzy R.; Vallone P. M.; Gallo F. J.; Paner T. M.; Lane M. J.; Benight A. S. Predicting sequence-dependent melting stability of short duplex DNA oligomers. Biopolymers 1997, 44, 217–239. 10.1002/(SICI)1097-0282(1997)44:3<217::AID-BIP3>3.0.CO;2-Y. 9591477

[ref121] Ouldridge T. E.; Louis A. A.; Doye J. P. Extracting bulk properties of self-assembling systems from small simulations. J. Phys.: Condens. Matter 2010, 22, 104102 10.1088/0953-8984/22/10/104102. 21389436

[ref122] Noé F.; Doose S.; Daidone I.; Löllmann M.; Sauer M.; Chodera J. D.; Smith J. C. Dynamical fingerprints for probing individual relaxation processes in biomolecular dynamics with simulations and kinetic experiments. Proc. Natl. Acad. Sci. U. S. A. 2011, 108, 4822–4827. 10.1073/pnas.1004646108. 21368203 PMC3064371

[ref123] Remington J. M.; McCullagh M.; Kohler B. Molecular Dynamics Simulations of 2-Aminopurine-Labeled Dinucleoside Monophosphates Reveal Multiscale Stacking Kinetics. J. Phys. Chem. B 2019, 123, 2291–2304. 10.1021/acs.jpcb.8b12172. 30767498

[ref124] Di Michele L.; Mognetti B. M.; Yanagishima T.; Varilly P.; Ruff Z.; Frenkel D.; Eiser E. Effect of Inert Tails on the Thermodynamics of DNA Hybridization. J. Am. Chem. Soc. 2014, 136, 6538–6541. 10.1021/ja500027v. 24750023

[ref125] Dickman R.; Manyanga F.; Brewood G. P.; Fish D. J.; Fish C. A.; Summers C.; Horne M. T.; Benight A. S. Thermodynamic Contributions of 5′- and 3′-Single Strand Dangling-Ends to the Stability of Short Duplex DNAs. J. Biophys. Chem. 2012, 3, 1–15. 10.4236/jbpc.2012.31001.

[ref126] Doktycz M. J.; Paner T. M.; Amaratunga M.; Benight A. S. Thermodynamic Stability of the 5 Dangling-Ended DNA Hairpins Formed from Sequences 5-(XY) 2GGATAC (T), GTATCC-3, Where X,Y = A,T,G,C. Biopolymers 1990, 30, 829–845. 10.1002/bip.360300718. 2275982

[ref127] Yakovchuk P.; Protozanova E.; Frank-Kamenetskii M. D. Base-Stacking and Base- Pairing Contributions into Thermal Stability of the DNA Double Helix. Nucleic Acids Res. 2006, 34, 564–574. 10.1093/nar/gkj454. 16449200 PMC1360284

[ref128] Zacharias M. Base-Pairing and Base-Stacking Contributions to Double-Stranded DNA Formation. J. Phys. Chem. B 2020, 124, 10345–10352. 10.1021/acs.jpcb.0c07670. 33156627

[ref129] McCully M. E.; Beck D. A.; Daggett V. Microscopic reversibility of protein folding in molecular dynamics simulations of the engrailed homeodomain. Biochemistry 2008, 47, 7079–7089. 10.1021/bi800118b. 18553935 PMC2905463

[ref130] Wu H.; Mey A. S.; Rosta E.; Noé F. Statistically optimal analysis of state-discretized trajectory data from multiple thermodynamic states. J. Chem. Phys. 2014, 141, 214106 10.1063/1.4902240. 25481128

