# Supplementary material for: Determining Sequence-Dependent DNA Oligonucleotide Hybridization and Dehybridization Mechanisms Using Coarse-Grained Molecular Simulation, Markov State Models, and Infrared Spectroscopy
Source: J Am Chem Soc. 2021 Oct 13;143(42):17395–411. doi: 10.1021/jacs.1c05219 (PMC8554761; doi:10.1021/jacs.1c05219)
Supplement: Supplementary file 1 — ja1c05219_si_001.pdf [file ja1c05219_si_001.pdf]

SUPPORTING INFORMATION

Determining sequence-dependent DNA oligonucleotide hybridization and dehybridization mechanisms using coarse-grained molecular simulation, Markov state models, and infrared spectroscopy

Michael S. Jones,† Brennan Ashwood,‡ Andrei Tokmakoff,‡ and Andrew L. Ferguson*,†

†*Pritzker School of Molecular Engineering, The University of Chicago, 5640 South Ellis Avenue, Chicago, Illinois 60637, United States*

‡*Department of Chemistry, Institute for Biophysical Dynamics, and James Franck Institute, The University of Chicago, 929 East 57th Street, Chicago, Illinois 60637, United States*

E-mail: andrewferguson@uchicago.edu

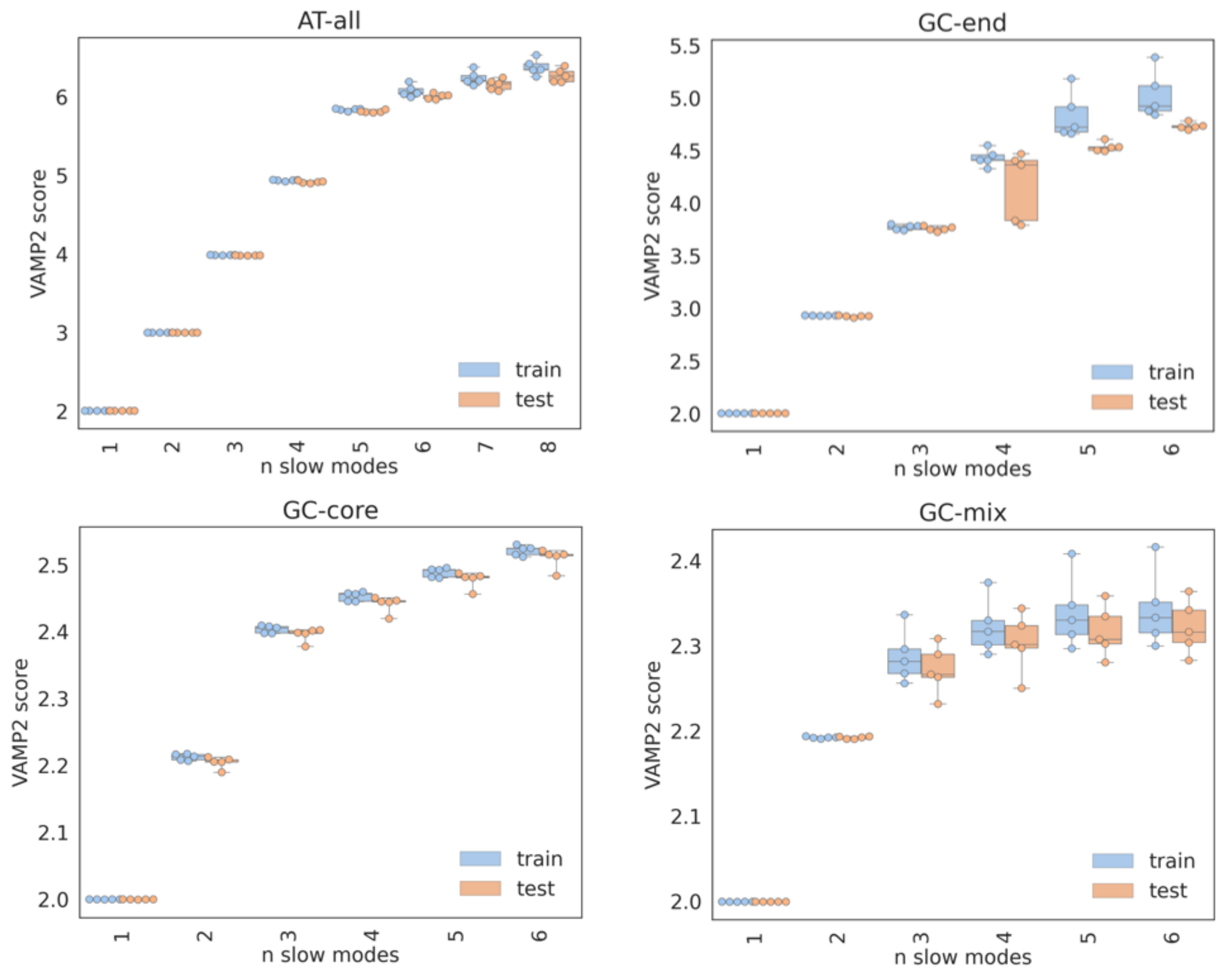

Figure S1: Five-fold cross-validation of the SRV VAMP-2 scores to select the optimal number of SRV coordinates for each sequence. A knee in the VAMP-2 plot was identified at the fifth, fourth, third, and second slow modes for AT-all, GC-end, GC-core, and GC-mix, respectively. An embedding of corresponding dimensionality was then used to cluster frames into discrete states. The absence of any significant separation in the training and testing VAMP-2 scores demonstrates that model is not overfitted.

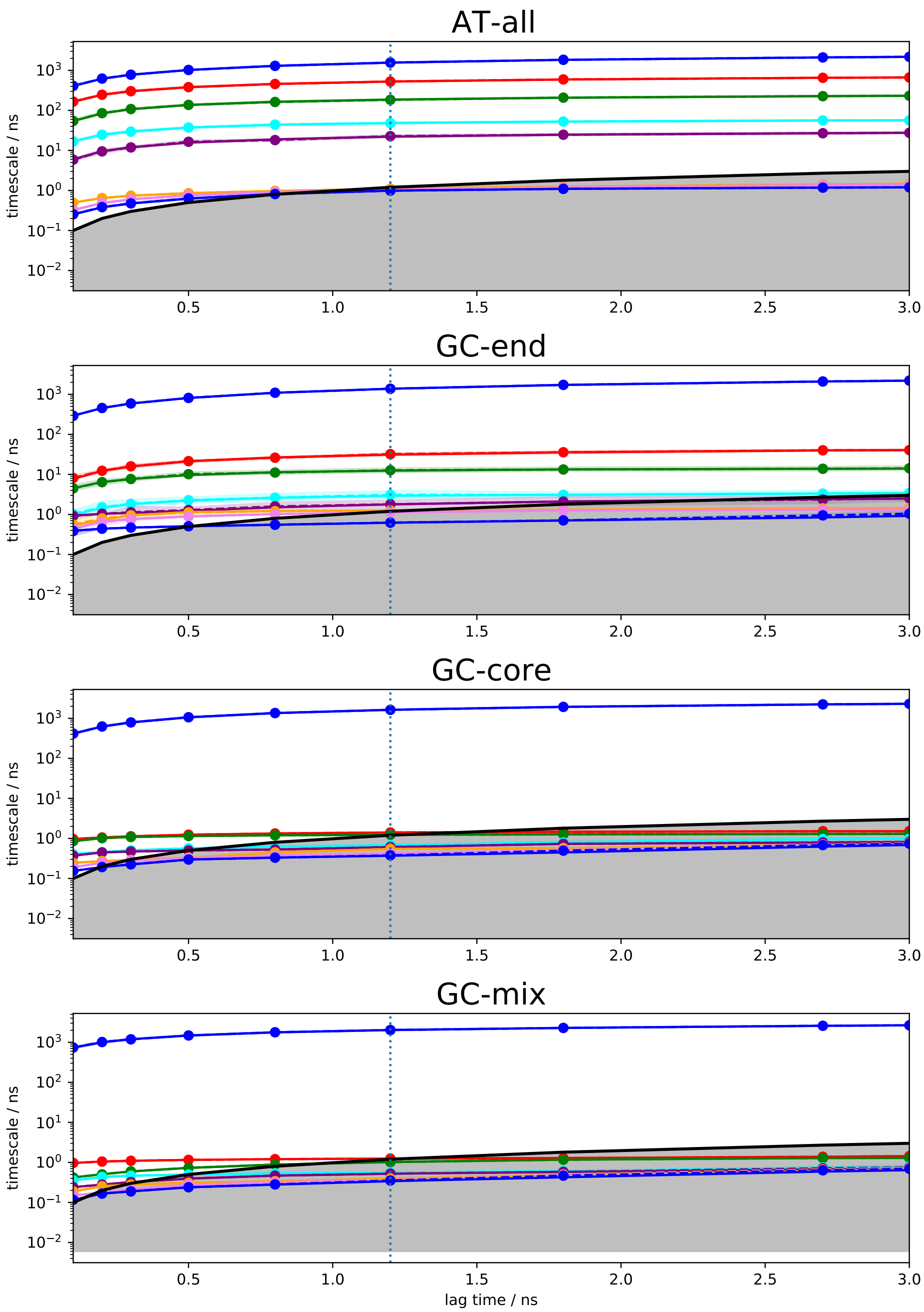

Figure S2: Convergence of the MSM implied time scales t_i as a function of lag time τ. Solid lines indicate maximum likelihood result while dashed lines show the Bayesian ensemble means superposed on shaded areas representing 95% confidence intervals. The high simulation data volume used in our MSM construction means that the maximum likelihood and Bayesian ensemble means are in good agreement and confidence intervals are narrow. The implied time scales for all sequences converge at a lag time of $\tau = 1.2$ ns (vertical line). The black solid curve marks equality of the implied time scale and lag time and delimits the shaded region wherein the implied time scales are shorter than the lag time and cannot be resolved.

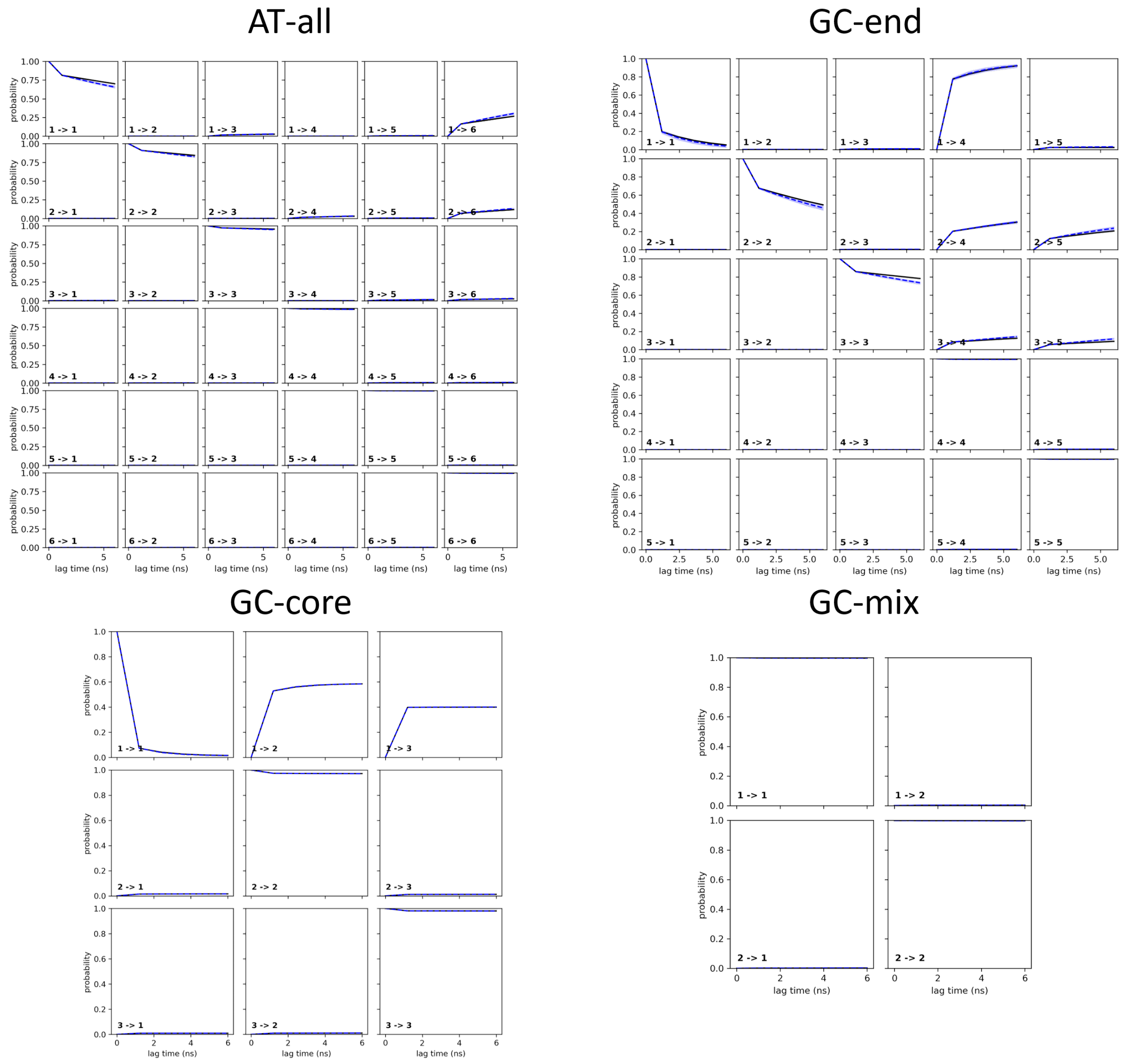

Figure S3: Chapman-Kolmogorov (CK) tests comparing the probabilities of remaining within each macrostates for each sequence as a function of lag time predicted by k applications of an MSM constructed at the $\tau = 1.2$ ns lag time $\mathbf{P}^k(\tau)$ (dashed blue line) versus those computed from an MSM constructed at that particular lag time $\mathbf{P}(k\tau)$ (solid black line). The blue shaded area indicates the estimated standard deviation in $\mathbf{P}^k(\tau)$. The good agreement between these two results provides numerical validation of the Markovian nature of the $\tau = 1.2$ ns lag time MSM.

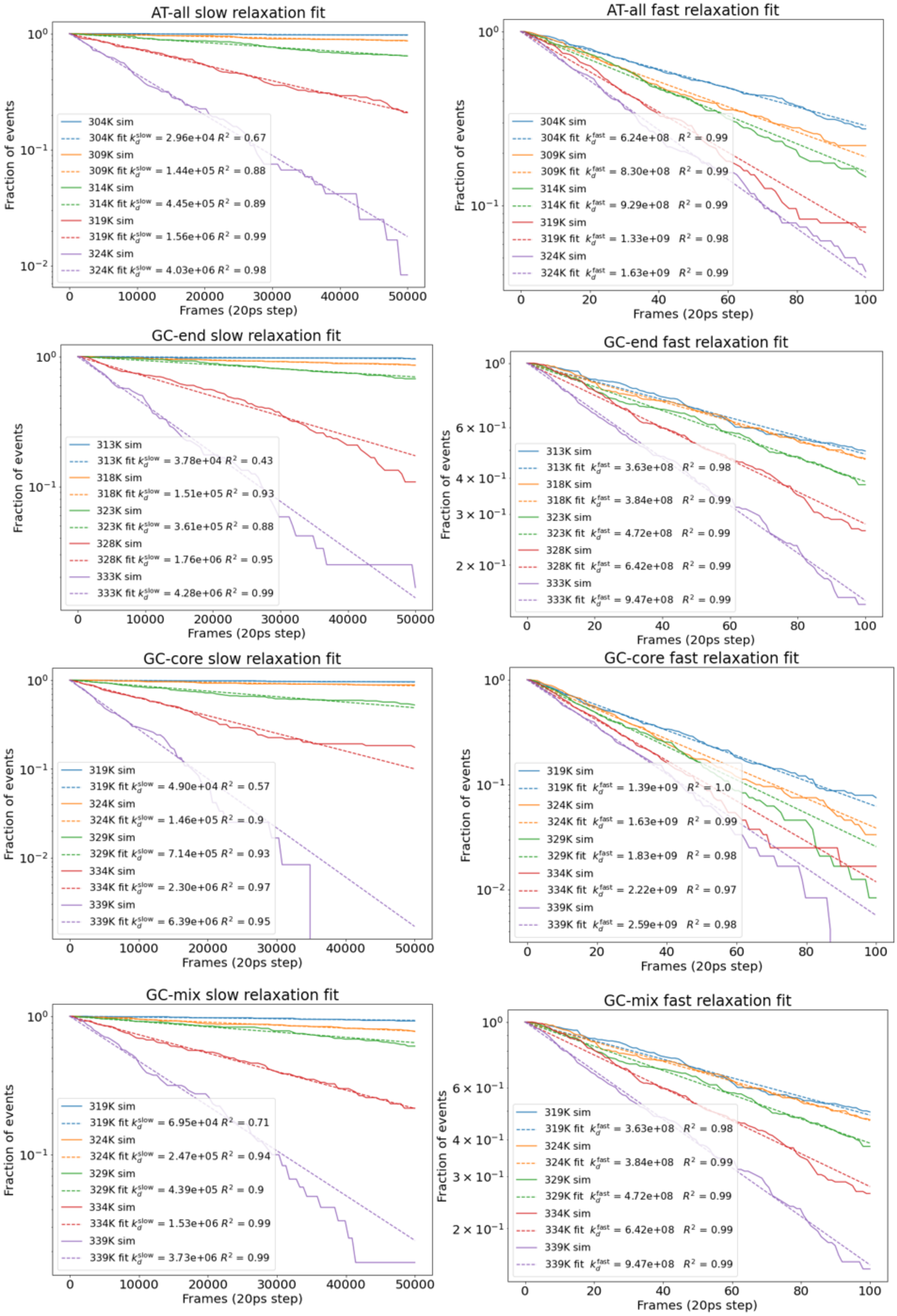
AT-all slow relaxation fit
Fraction of events
Frames (20ps step)
304K sim
304K fit k_d^{slow} = 2.96e+04 R^2 = 0.67
309K sim
309K fit k_d^{slow} = 1.44e+05 R^2 = 0.88
314K sim
314K fit k_d^{slow} = 4.45e+05 R^2 = 0.89
319K sim
319K fit k_d^{slow} = 1.56e+06 R^2 = 0.99
324K sim
324K fit k_d^{slow} = 4.03e+06 R^2 = 0.98
AT-all fast relaxation fit
Fraction of events
Frames (20ps step)
304K sim
304K fit k_d^{fast} = 6.24e+08 R^2 = 0.99
309K sim
309K fit k_d^{fast} = 8.30e+08 R^2 = 0.99
314K sim
314K fit k_d^{fast} = 9.29e+08 R^2 = 0.99
319K sim
319K fit k_d^{fast} = 1.33e+09 R^2 = 0.98
324K sim
324K fit k_d^{fast} = 1.63e+09 R^2 = 0.99
GC-end slow relaxation fit
Fraction of events
Frames (20ps step)
313K sim
313K fit k_d^{slow} = 3.78e+04 R^2 = 0.43
318K sim
318K fit k_d^{slow} = 1.51e+05 R^2 = 0.93
323K sim
323K fit k_d^{slow} = 3.61e+05 R^2 = 0.88
328K sim
328K fit k_d^{slow} = 1.76e+06 R^2 = 0.95
333K sim
333K fit k_d^{slow} = 4.28e+06 R^2 = 0.99
GC-end fast relaxation fit
Fraction of events
Frames (20ps step)
313K sim
313K fit k_d^{fast} = 3.63e+08 R^2 = 0.98
318K sim
318K fit k_d^{fast} = 3.84e+08 R^2 = 0.99
323K sim
323K fit k_d^{fast} = 4.72e+08 R^2 = 0.99
328K sim
328K fit k_d^{fast} = 6.42e+08 R^2 = 0.99
333K sim
333K fit k_d^{fast} = 9.47e+08 R^2 = 0.99
GC-core slow relaxation fit
Fraction of events
Frames (20ps step)
319K sim
319K fit k_d^{slow} = 4.90e+04 R^2 = 0.57
324K sim
324K fit k_d^{slow} = 1.46e+05 R^2 = 0.9
329K sim
329K fit k_d^{slow} = 7.14e+05 R^2 = 0.93
334K sim
334K fit k_d^{slow} = 2.30e+06 R^2 = 0.97
339K sim
339K fit k_d^{slow} = 6.39e+06 R^2 = 0.95
GC-core fast relaxation fit
Fraction of events
Frames (20ps step)
319K sim
319K fit k_d^{fast} = 1.39e+09 R^2 = 1.0
324K sim
324K fit k_d^{fast} = 1.63e+09 R^2 = 0.99
329K sim
329K fit k_d^{fast} = 1.83e+09 R^2 = 0.98
334K sim
334K fit k_d^{fast} = 2.22e+09 R^2 = 0.97
339K sim
339K fit k_d^{fast} = 2.59e+09 R^2 = 0.98
GC-mix slow relaxation fit
Fraction of events
Frames (20ps step)
319K sim
319K fit k_d^{slow} = 6.95e+04 R^2 = 0.71
324K sim
324K fit k_d^{slow} = 2.47e+05 R^2 = 0.94
329K sim
329K fit k_d^{slow} = 4.39e+05 R^2 = 0.9
334K sim
334K fit k_d^{slow} = 1.53e+06 R^2 = 0.99
339K sim
339K fit k_d^{slow} = 3.73e+06 R^2 = 0.99
GC-mix fast relaxation fit
Fraction of events
Frames (20ps step)
319K sim
319K fit k_d^{fast} = 3.63e+08 R^2 = 0.98
324K sim
324K fit k_d^{fast} = 3.84e+08 R^2 = 0.99
329K sim
329K fit k_d^{fast} = 4.72e+08 R^2 = 0.99
334K sim
334K fit k_d^{fast} = 6.42e+08 R^2 = 0.99
339K sim
339K fit k_d^{fast} = 9.47e+08 R^2 = 0.99

Figure S4: Exponential fits for both slow (dissociation) and fast (fraying) response for all four sequences during "computational T-jump" experiments. From 120 independent 1 μs simulations, we compiled the slow response data by recording the fraction of sequences with both central Watson-Crick base pairs intact as a function of time, and the fast response data as the fraction of sequences with both terminal Watson-Crick base pairs intact as a function of time. We define a Watson-Crick base pair to be intact if the centers-of-mass of the two comlementary bases lie within a linear distance of 1.3 nm. We extracted our computational estimate of k_d^{fast} by fitting a decaying exponential to the fraction of bound A:T termini as a function of time $f_{\text{unfrayed}}(t) = \exp(-k_d^{\text{fast}} t)$. Similarly, we extracted our computational estimate of k_d^{slow} by fitting a decaying exponential to the fraction of hybridized sequences as a function of time $f_{\text{hybridized}}(t) = \exp(-k_d^{\text{slow}} t)$. We report within the figure legend of each panel the coefficient of determination R^2 for a least squares linear fit of the model to the data in log space (i.e., $\log(f) = -k_d t$) and data are plotted on log-linear axes to facilitate visual comparison of the fits. In all cases we observe excellent fits of the models to the data with all $R^2 > 0.88$ except for the slow response at the the lowest temperature T_m - 5 K where dissociation events are sparse.

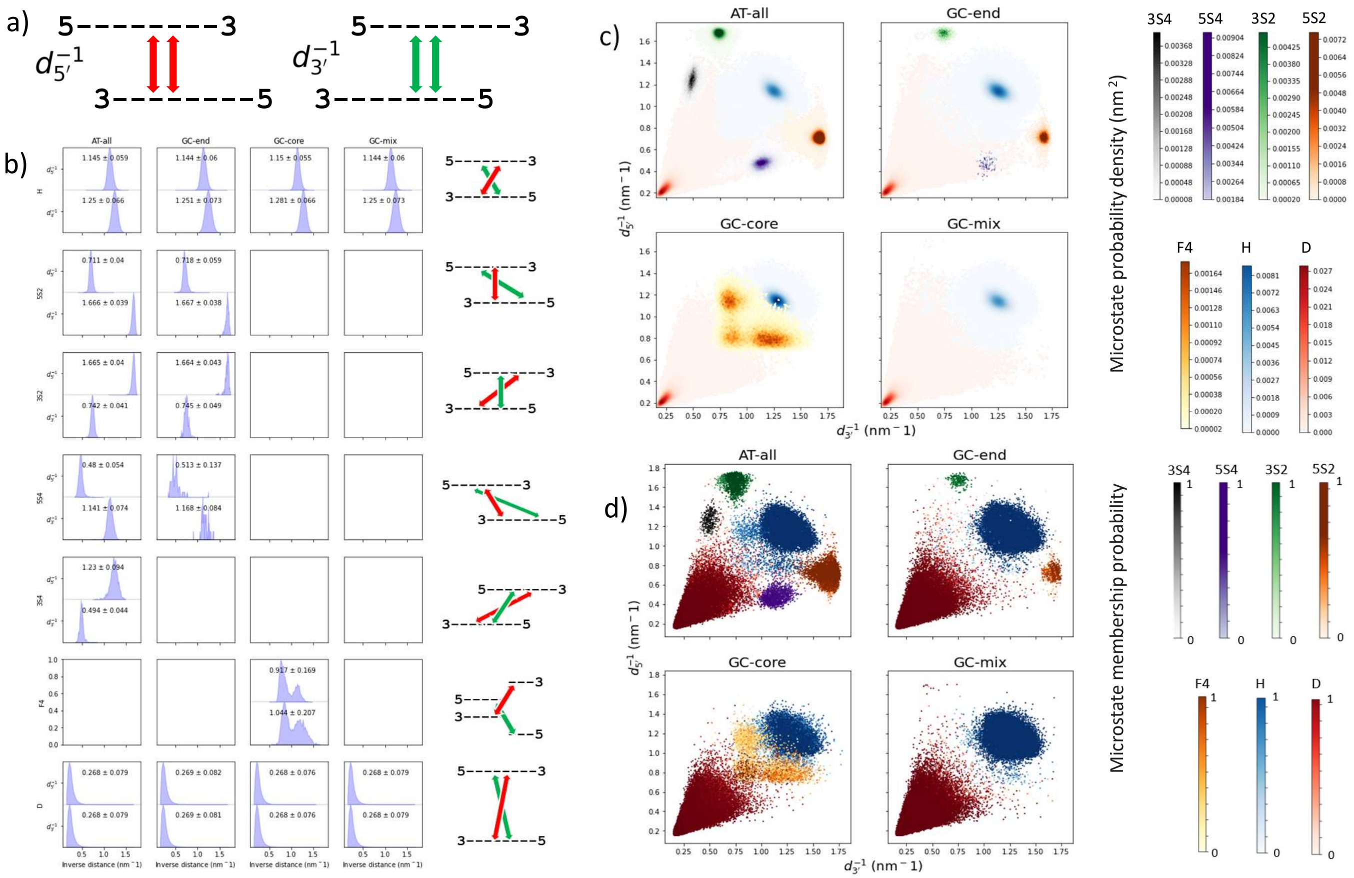
a)
b)
c)
d)
AT-all
GC-end
GC-core
GC-mix
1.145 ± 0.059
1.25 ± 0.066
1.144 ± 0.06
1.251 ± 0.073
1.15 ± 0.055
1.281 ± 0.066
1.144 ± 0.06
1.25 ± 0.073
0.711 ± 0.04
1.666 ± 0.039
0.718 ± 0.059
1.667 ± 0.038
1.665 ± 0.04
0.742 ± 0.041
1.664 ± 0.043
0.745 ± 0.049
0.48 ± 0.054
1.141 ± 0.074
0.513 ± 0.137
1.168 ± 0.084
1.23 ± 0.094
0.494 ± 0.044
0.917 ± 0.169
1.044 ± 0.207
0.268 ± 0.079
0.268 ± 0.079
0.269 ± 0.082
0.269 ± 0.081
0.268 ± 0.076
0.268 ± 0.076
0.268 ± 0.079
0.268 ± 0.079
Inverse distance (nm⁻¹)
Microstate probability density (nm²)
Microstate membership probability
3S4
5S4
3S2
5S2
F4
H
D

Figure S5: Macrostate interpretation and structural analysis. The Markov state model macrostates are learned from the simulation trajectories using the featurization and deep learning procedure described in Section 2.1.2 of the main text, but it is informative to perform a *post hoc* projection of the simulation data into a low-dimensional space spanned by physically interpretable order parameters in order to determine the structural characteristics of each macrostate and the structural heterogeneity of the microstate ensemble. (a) Definition of two physically-interpretable order parameters useful in characterizing the structure and base pairing of each microstate under both shifting and fraying. We define the $3'$ shift for a particular base on a strand as the linear distance between that base and the base on the complementary strand offset by two base pairs in the $3'$ direction. This distance reaches a minimum when the two strands are mutually shifted two base pairs in the $3'$ direction. We define $d_{3'}$ as the mean $3'$ shift averaged over the two central bases, represented by red arrows, in the complementary strands. It is more convenient to work the reciprocal $d_{3'}^{-1}$, since it has a finite range whereas $d_{3'}$ diverges for dehybridized strands. We define $d_{5'}^{-1}$ analogously as the reciprocal mean $5'$ shift, represented by green arrows. We have found this pair of variables extremely informative in revealing the microstate and macrostate structure and base pairing patterns. (b) We report the mean and standard deviation of $d_{3'}$ and $d_{5'}$ taken over all microstates comprising each macrostate for each sequence. The small standard deviations that are consistent with a narrow distribution of the bulk of the microstates around the free energy minimum of each macrostate. Cartoon renderings reflect the dominant configurations of each macrostate and how each is represented by the $d_{3'}^{-1}$ and $d_{5'}^{-1}$ order parameters green and red arrows, respectively. (c) Superposition of sample density for each macrostate for each sequence AT-all, GC-end, GC-core, and GC-mix projected into $d_{3'}^{-1}$-$d_{5'}^{-1}$. This panel conveys two important pieces of information. First, each macrostate is anchored around a single free energy minimum containing the preponderance of the probability mass of the microstate ensemble. These projections indicate a relatively narrowly peaked distribution of microstates around the free energy minimum with a broad tail. Second, the location of each macrostate free energy minimum in the $d_{3'}^{-1}$-$d_{5'}^{-1}$ projection readily illuminates the base pair pattern within the duplex. Within the fully hybridized H state (blue) $d_{3'}^{-1} \approx d_{5'}^{-1} \approx$ 1.2 nm^{-1}. A 2 b.p. shift to 3′ is manifest in a decrease in $d_{3'}^{-1}$ (increase in $d_{3'}$) and an increase in $d_{5'}^{-1}$ (decrease in $d_{5'}$) producing the stable 3S2 state (green). A further 2 b.p. shift to 3′ results in a further decrease in $d_{3'}^{-1}$ and also a decrease in $d_{5'}^{-1}$ (back down to the same value as in the H state) to produce the 3S4 state (black). Analogous arguments apply to the 2 b.p. 5′ shifted state 5S2 (orange) and 4 b.p. 5′ shifted state 5S4 (purple). The dehybridized state D exists, due to the finite box size, at $d_{3'}^{-1} \approx d_{5'}^{-1} \approx$ 0.2 nm^{-1} (red). The GC-core frayed state F4 (orange) exists as a heart-shaped region between the H and D sates, with each symmetric lobe of the heart corresponding to fraying of one or other of the loose ends. (d) Coloring the microstates by their membership probabilities to their assigned macrostate under a fuzzy clustering procedure shows that microstates lying in the vicinity of the free energy minima have high probabilities whereas those at the macrostate cluster boundaries have less definitive assignments. The former comprise the preponderance of the microstate ensemble while the latter represent intermediate configurations that are transiently occupied as the system transitions from one macrostate to another.

Nearest neighbor model of duplex thermodynamics

DNA duplex hybridization thermodynamics are commonly predicted using nearest-neighbor (NN) models wherein the stability of a given base pair is assumed to solely depend on the identity and orientation of its adjacent base pairs. We apply the popular NN model reported by SantaLucia[1] to estimate the stability of fully intact, shifted, and frayed duplex configurations observed in our MSMs.

The NN model assumes hybridization proceeds in a two-state all-or-nothing manner. The Gibbs free energy difference between the hybridized and dissociated states can be determined from calculation of NN enthalpy and entropy contributions,

$$\Delta G^{\circ}_{NN}(T) = \Delta H^{\circ}_{NN} - T\Delta S^{\circ}_{NN}. \tag{1}$$

ΔH°_{NN} and ΔS°_{NN} are assumed to be independent of temperature T, and are computed from the sum over all NN terms in a given configuration,

$$\Delta H^{\circ}_{NN} = \sum_{i}^{n_{NN}} \Delta H^{\circ}_{NN,i} + \sum_{j}^{n_{DE}} \Delta H^{\circ}_{DE,j} + \Delta H^{\circ}_{init}, \tag{2}$$

$$\Delta S^{\circ}_{NN} = \sum_{i}^{n_{NN}} \Delta S^{\circ}_{NN,i} + \sum_{j}^{n_{DE}} \Delta S^{\circ}_{DE,j} + \Delta S^{\circ}_{init} + \Delta S^{\circ}_{sym}. \tag{3}$$

$\Delta H^{\circ}_{NN,i}$ is the NN enthalpy for a given dinucleotide step within duplex DNA, $\Delta H^{\circ}_{DE,j}$ corresponds to the enthalpic contribution from a given dangling end (DE) base next to the duplex[2], and ΔH°_{init} is a duplex initiation term that accounts for terminal effects on duplex enthalpy. A schematic illustration of the NN and DE contributions for a fully hybridized duplex and an out-of-register shifted state is presented in Fig. S6. ΔS°_{NN} is constructed in an analogous manner from NN, dangling end, and initiation terms, plus a symmetry term, ΔS°_{sym}, that applies an entropic penalty to self-complementary sequences for maintaining C2 symmetry.

The SantaLucia NN parameters are defined for a solution condition at neutral pH with a sodium concentration of 1M. We use the empirical salt correction developed by Owczarzy[3] to determine the NN parameters at the sodium concentration used for simulations in this work. The Owczarzy model assumes the effect of counter-ions on DNA hybridization thermodynamics to be purely entropic,

$$\Delta S^{\circ}_{NN}(c_{Na^+}) = S^{\circ}_{NN}(1M) + \Delta H^{\circ}_{NN}[(4.29 f_{GC} - 3.95) \times 10^{-5} \ln(c_{Na^+}) + 9.4 \times 10^{-6} (\ln(c_{Na^+}))^2], \quad (4)$$

where f_{GC} is the fractional G:C content of the duplex and c_{Na^+} is the concentration of sodium counter-ions measured in M.

In applying the NN model to each macrostate, we made the simplifying assumption that the ensemble of microstates constituting each macrostate could be represented by a single pattern of Watson-Crick base pairing that are schematically illustrated in Fig. 2a. In reality, each macrostate is composed of an ensemble of microstates that may have slightly different base pairing patterns and explicitly averaging over all of these microstates may change the predictions of the NN model. To evaluate the extent to which the microstate ensembles would change our free energy estimates, we calculated a NN microstate free energy by taking a simple average over the NN free energies predicted for each configuration within a given microstate – a reasonable assumption given the structural similarity of configurations within a microstate – then computing macrostate free energies via a weighted average according to the MSM equilibrium occupancy probabilities for each microstate. These operations can be represented as follows, where i indexes over the N_j configurations in microstate j, j indexes over microstates, and p_j represents the MSM equilibrium occupancy of microstate j,

$$F^{micro} = \frac{1}{N_j} \sum_{i \in micro} F_i^{NN} \quad (5)$$

$$F^{macro} = \sum_{j \in macro} p_j F_j^{micro} \quad (6)$$

Recalculating the NN predictions in this way leads to changes in the $\Delta F = F - F_H$ values reported in Fig. 3 (i.e., the free energy of each macrostate relative to the hybridized state) of <1 kJ/mol. As such, representing each macrostate by a single representative microstate and base pairing pattern is a highly accurate simplifying approximation for the purposes of this calculation.

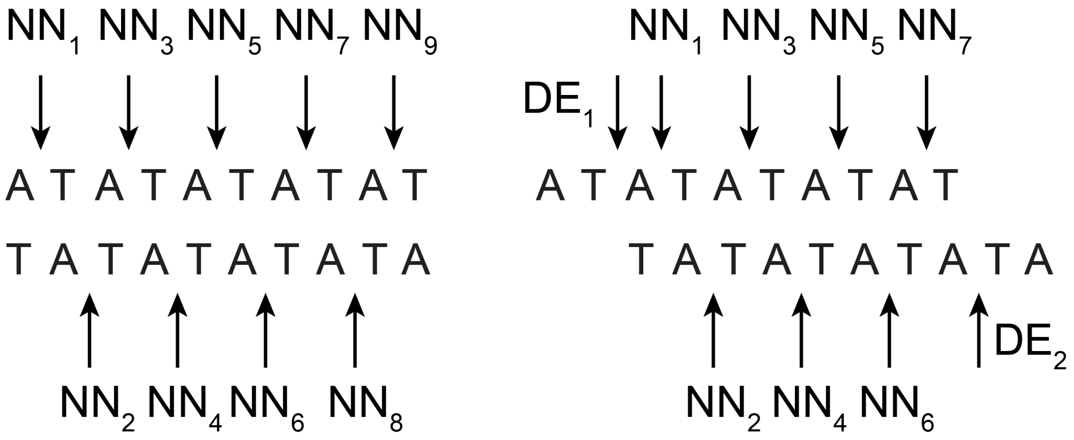

Figure S6: Schematic of nearest neighbor (NN) contributions for a fully hybridized AT-all duplex and an out-of-register duplex with dangling ends (DE).

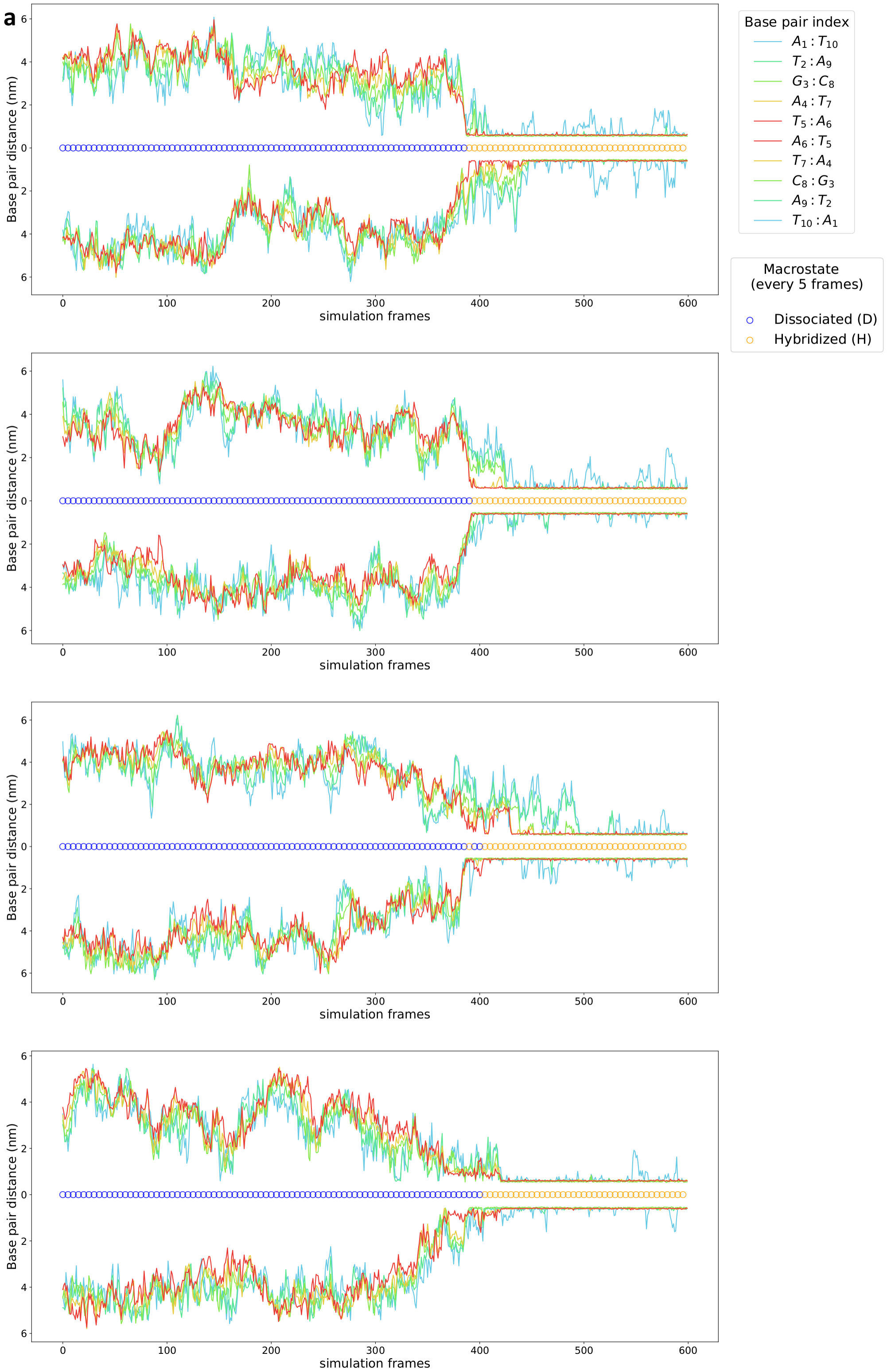
a
Base pair distance (nm)
simulation frames
Base pair index
$A_1 : T_{10}$
$T_2 : A_9$
$G_3 : C_8$
$A_4 : T_7$
$T_5 : A_6$
$A_6 : T_5$
$T_7 : A_4$
$C_8 : G_3$
$A_9 : T_2$
$T_{10} : A_1$
Macrostate
(every 5 frames)
Dissociated (D)
Hybridized (H)

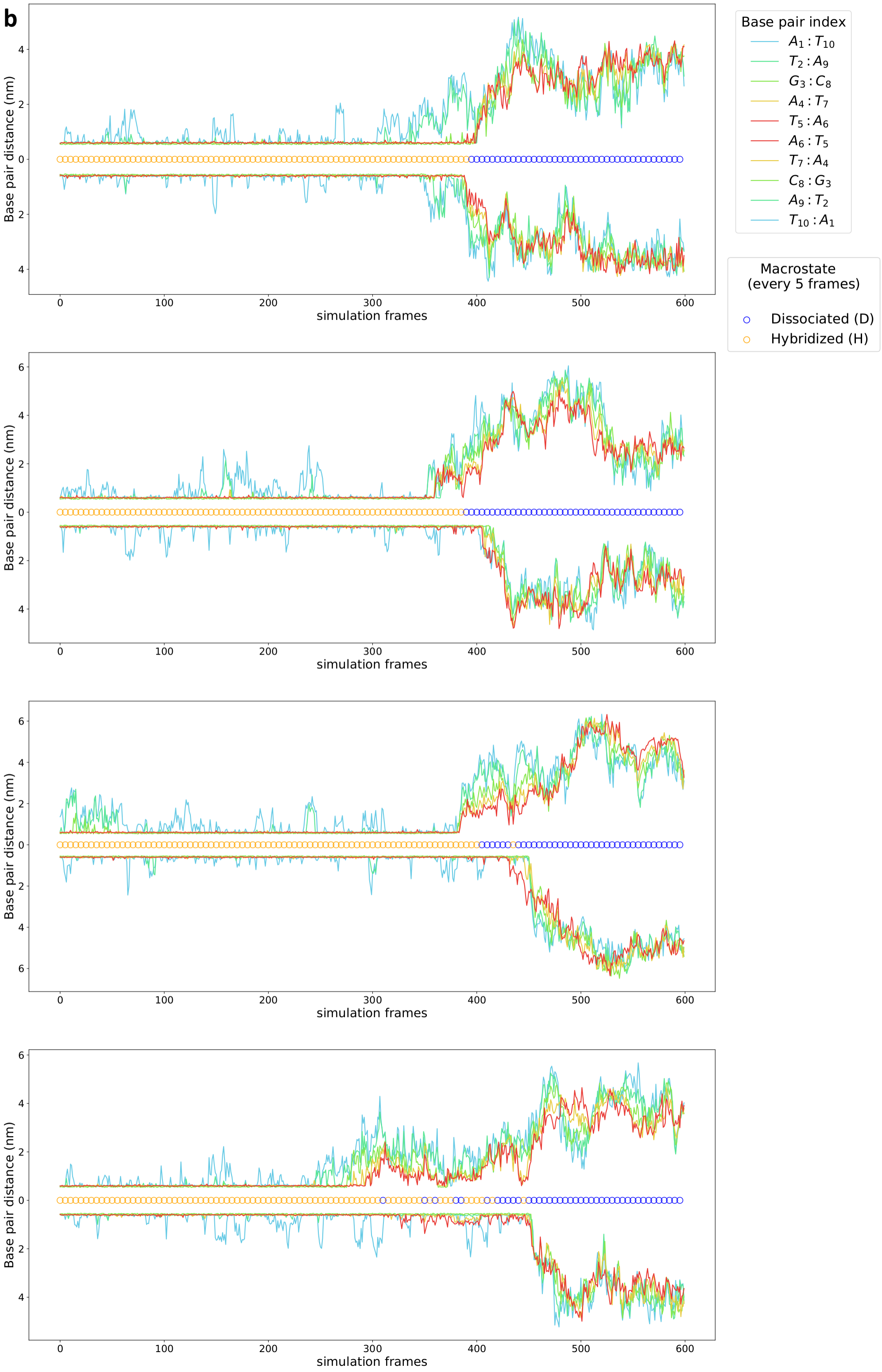
b
Base pair index
$A_1 : T_{10}$
$T_2 : A_9$
$G_3 : C_8$
$A_4 : T_7$
$T_5 : A_6$
$A_6 : T_5$
$T_7 : A_4$
$C_8 : G_3$
$A_9 : T_2$
$T_{10} : A_1$
Macrostate (every 5 frames)
Dissociated (D)
Hybridized (H)
Base pair distance (nm)
simulation frames

Figure S7: GC-mix hybridizes by nucleation-zippering and dehybridizes by fraying-peeling. Tracking of the 10 intermolecular distances between native WC base pairs over the course of four additional (a) hybridization and (b) dehybridization events. Symmetrically permutable distances (e.g., A_1:T_{10} and T_{10}:A_1) are reflected across the x-axis to avoid congestion in the plot. Circles superposed on the x-axis indicate the instantaneous MSM state assignment as dissociated D (blue) or hybridized H (orange). Hybridization tends to occur by a nucleation-zippering mechanism, wherein a native G:C pair and adjacent A:T pair or 2-3 central A:T pairs first form prior to rapid formation of the duplex. Dehybridization tends to occur by a fraying-peeling mechanism wherein fraying of the two-base AT-tails on one or both sides of the duplex precedes dissociation of the central native base pairs and complete dissolution of the duplex.

References

(1) SantaLucia, J. A Unified View of Polymer, Dumbbell, and Oligonucleotide DNA Nearest-Neighbor Thermodynamics. *Proc. Natl. Acad. Sci. U. S. A.* **1998**, *95*, 1460–1465.

(2) Santalucia, J.; Hicks, D. The Thermodynamics of DNA Structural Motifs. *Annu. Rev. Biophys. Biomol. Struct* **2004**, *33*, 415–440.

(3) Owczarzy, R.; Moreira, B. G.; You, Y.; Behlke, M. A.; Wälder, J. A. Predicting Stability of DNA Duplexes in Solutions Containing Magnesium and Monovalent Cations. *Biochemistry* **2008**, *47*, 5336–5353.
